# Threefold increase in atmospheric–riverine compound heatwaves under climate change

**DOI:** 10.1038/s41561-026-02040-y

**Published:** 2026-07-30

**Authors:** Yu Zhou, Yanxia Shen, Wei Zhi, Bo Zhou, Senlin Zhu, Li Yuan, Yangyang Wang, Chuan Wang, Kun Shi, Amir Aghakouchak, Michelle T. H. van Vliet, Chunhui Lu, R. Iestyn Woolway

**Affiliations:** 1 State Key Laboratory of Water Disaster Prevention, Yangtze Institute for Conservation and Development, Hohai University, Nanjing, China; 2 Key Laboratory of Hydrologic-Cycle and Hydrodynamic-System of Ministry of Water Resources, College of Hydrology and Water Resources, Hohai University, Nanjing, China; 3 Bureau of Hydrology, Changjiang Water Resources Commission, Ministry of Water Resources, Wuhan, China; 4 College of Hydraulic Science and Engineering, Yangzhou University, Yangzhou, China; 5 State Key Laboratory of Advanced Environmental Technology, Department of Environmental Science and Engineering, University of Science and Technology of China, Hefei, China; 6 Key Laboratory of Lake and Watershed Science for Water Security, Nanjing Institute of Geography and Limnology, Chinese Academy of Sciences, Nanjing, China; 7 Department of Civil and Environmental Engineering, University of California, Irvine, CA USA; 8 Department of Physical Geography, Utrecht University, Utrecht, the Netherlands; 9 School of Ocean Sciences, Bangor University, Bangor, UK

**Keywords:** Hydrology, Natural hazards, Projection and prediction

## Abstract

Record-breaking heatwaves disrupt global water, energy and food systems, yet the co-occurrence of atmospheric and riverine events remains largely unexplored. Here we analyse 796 river basins in the USA and Central Europe to characterize such co-occurring atmospheric–riverine compound heatwaves. Combining water quality observations with a deep learning model, we find that compound heatwaves have increased by about 0.40 events per decade since the 1980s. This corresponds to a rise from roughly 0.76 events per year in the early period to about three times that frequency today, meaning their occurrence has effectively tripled over the past four decades. This trend coincides with the rapid intensification of riverine heatwaves, which have increased in frequency (114%), duration (148%) and intensity (95%) between 1981–1990 and 2010–2019, far outpacing changes in atmospheric heatwaves. Compound occurrences are primarily controlled by climatic (59.2%), topographic (22.4%) and hydrological (18.3%) factors, with amplified trends in high-elevation (>3,000 m) mountain rivers (+128% per decade). Compared with isolated riverine heatwaves, compound heatwaves drive an additional 16% rise in water temperature and a further 2.9% decline in dissolved oxygen. Under a high-emissions scenario, 98.5% of riverine heatwaves will coincide with atmospheric heatwaves by 2100. These findings highlight the escalating threats of compound heatwaves to freshwater ecosystems and the need to incorporate their dynamics into future water risk assessments.

## Abstract (web summary)

Co-occurring atmospheric and river heatwaves have tripled since the 1980s across the USA and Europe, and are projected to become the norm by 2100, according to model simulations.

## Main

Global temperature records continue to be broken, intensifying heatwave risks to public health, energy infrastructure and global economies^1^. Atmospheric heatwaves (AHWs), defined as multi-day extreme events of abnormally high temperatures relative to local climatology, are becoming more frequent, longer lasting and more intense worldwide^2,3^. In the USA, the average number of heatwaves in major cities has tripled since the 1960s^4^, while Europe is warming three to four times faster compared with the rest of the northern mid-latitudes^5^. Recent evidence, including the intense 2024 heatwave across the western USA^6^, illustrates the growing potential of AHWs to elevate wildfire risk and amplify social and economic impacts^7^. As climate warming continues, AHWs are projected to further escalate^8,9^, making heatwaves a heightened feature of future climate risk.

Rivers mediate hydro–biogeochemical exchanges between the atmosphere and land and are highly sensitive to climate change^10,11^. Excessive river warming can threaten aquatic life^12^, disrupt ecosystem metabolism^13^ and exacerbate chemical pollution^14^. Yet, compared with the well-studied marine^15,16^ and lake^17,18^ heatwaves, riverine heatwaves (RHWs) have received far less attention, even though rivers are shallow, fast-flowing and short-residence systems whose temperature can be more episodic and responsive to local weather. This owes in part to the scarcity and discontinuity of long-term, large-scale river water quality data^19^. Recently, emerging evidence suggests that RHWs are increasing rapidly and may outpace AHWs in some regions^20–22^. However, the extent to which RHWs co-occur with AHWs, and the mechanisms controlling these compound events, remain unclear.

**Extended Data Fig. 1:**
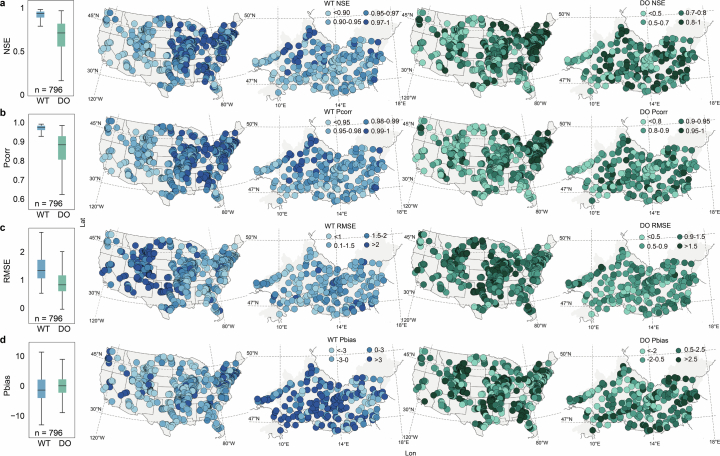
Testing performance of the LSTM model for WT and DO in the US and CE. **a–d**, Testing NSE, Pcorr, RMSE and Pbias, respectively. Left boxplots show basin-level distributions for WT and DO across 796 basins (n); boxplots indicate the median, interquartile range, and 1.5×IQR whiskers. Maps show WT performance in the US and CE, followed by corresponding DO performance. Circle colour indicates metric magnitude; blue and green denote WT and DO. Maps generated with US boundaries data from the US Census Bureau and Central European country boundaries data from Eurostat/GISCO Countries 2020, EuroGeographics.

**Extended Data Fig. 2:**
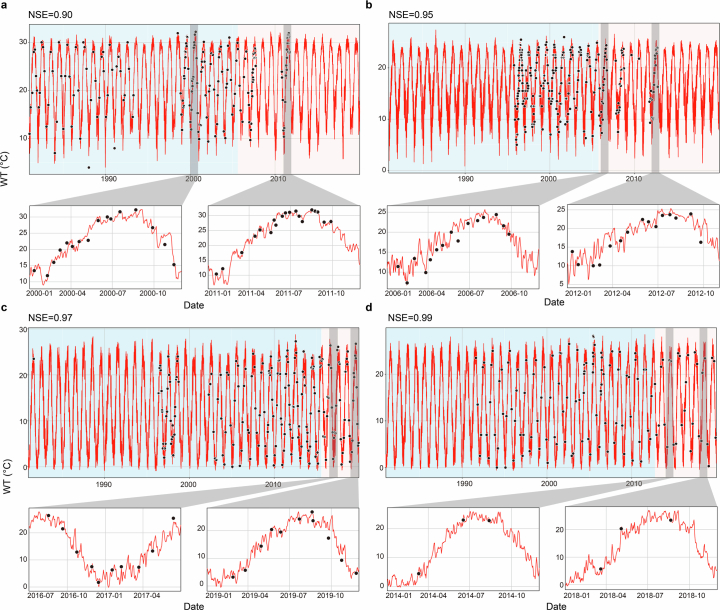
Representative LSTM simulations of daily WT. **a–d**, Observed and simulated daily WT for four representative basins with testing NSE values corresponding to the 25^th^, 50^th^, 75^th^ and 100^th^ percentiles, respectively. Black dots indicate observations and red lines indicate LSTM simulations. Blue and pink shading denote training and testing periods, respectively; grey shading marks periods enlarged below to show model performance during extreme WT conditions.

This knowledge gap matters because river ecosystems are vulnerable to multiple stressors^23^. When AHWs and RHWs co-occur in close proximity, their combined effects may exceed those of isolated events, intensifying thermal stress and threatening aquatic ecosystems^24^. Overlooking these compound processes may therefore underestimate freshwater risks and weaken the scientific basis for watershed management and adaptation planning^25^. Here we ask the following questions: (1) What are the spatiotemporal trends and main drivers of atmospheric–riverine compound heatwaves (ARCHs)? and (2) How do ARCH events impact river water quality compared with isolated heatwaves? We develop a continental-scale deep learning model to overcome the challenge of data scarcity, reconstructing continuous daily records (1981–2019) across 796 rivers in the USA and Central Europe (CE; Extended Data Figs. 1 and 2). We then analyse the spatiotemporal trends and dominant drivers of ARCHs, assess their impact on water quality and project future changes under different climate scenarios.

## Rising riverine and compound heatwaves

**Fig. 1:**
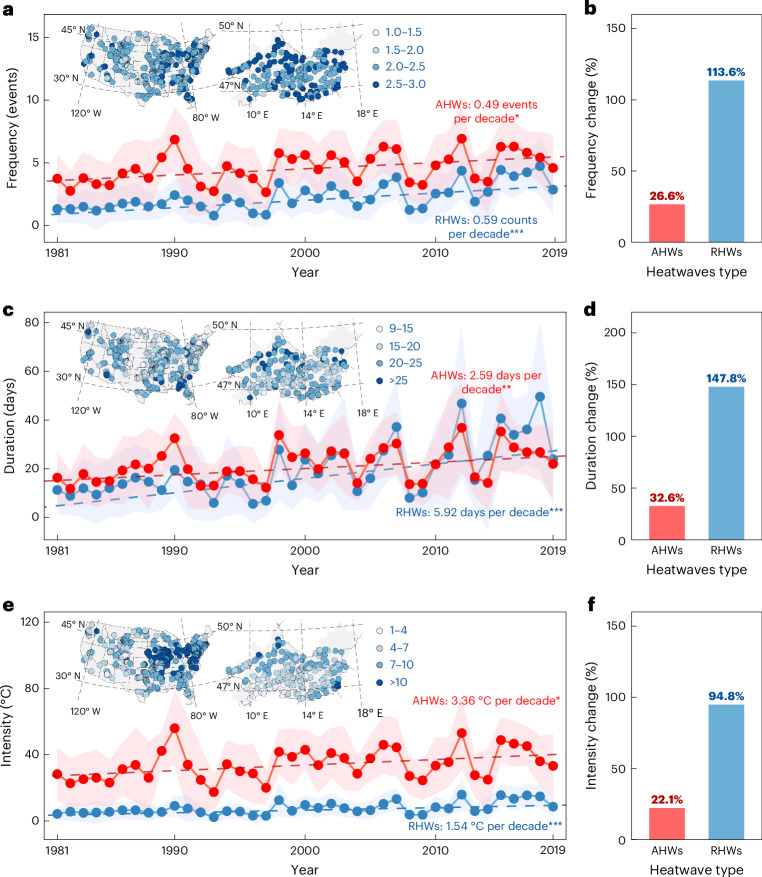
Trends in AHWs and RHWs across 796 river basins from 1981 to 2019. **a**,**b**, Annual mean frequency (**a**) and its percentage change (**b**) in 2010–2019 relative to 1981–1990. **c**,**d**, Annual mean duration (**c**) and percentage change (**d**). **e**,**f**, Annual mean intensity (**e**) and percentage change (**f**). Dots show basin means and shading indicates ± s.d. Inset maps show the spatial distributions of mean annual RHW metrics. Trends and significance were estimated using Theil–Sen analysis. Significance (two-sided test): *P < 0.05, **P < 0.01, ***P < 0.001. Maps in **a**,**c**,**e** generated with US boundaries data from the US Census Bureau and Central European country boundaries data from Eurostat/GISCO Countries 2020, EuroGeographics.

**Extended Data Fig. 3:**
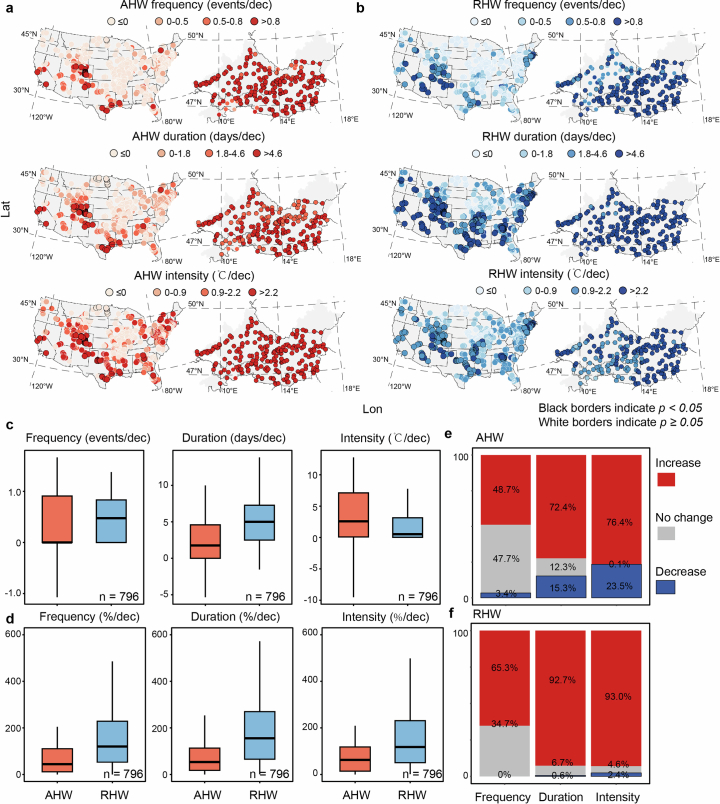
Basin-level changes in AHW and RHW metrics, 1981–2019. **a, b**, Maps of absolute changes in AHW and RHW frequency, duration and intensity across 796 basins in the US and CE; black and white borders indicate significant and non-significant trends, respectively. **c, d**, Basin-level distributions of absolute and percentage changes in frequency, duration and intensity for AHWs and RHWs. Box centre lines show medians, bounds 25^th^/75^th^ percentiles, and whiskers minima/maxima within 1.5× IQR. **e, f**, Fractions of basins showing increasing, decreasing or non-significant trends in AHW and RHW metrics. Maps in **a** and **b** generated with US boundaries data from the US Census Bureau and Central European country boundaries data from Eurostat/GISCO Countries 2020, EuroGeographics.

Our analysis across 796 river basins reveals that both AHWs and RHWs have intensified over the past 40 years. Notably, RHWs exhibited greater increase than AHWs in frequency and duration (Fig. 1a,c). RHW frequency increased by 0.59 events per decade, exceeding the increase of 0.49 events per decade in AHWs. This rate is comparable to previous estimates for US rivers^21^ and faster than that reported for marine heatwaves^26^. RHW duration increased by 5.92 days per decade, more than twice the rate of AHWs. Although RHWs exhibited smaller absolute increases in intensity than AHWs, their relative increases in frequency (113.6%), duration (147.8%) and intensity (94.8%) between 1981–1990 and 2010–2019 were larger than those of AHWs (26.6%, 32.6% and 22.1%, respectively; Fig. 1b,d,f). These results remain robust to alternative heatwave definitions (Methods and Extended Data Table 1). Spatial analysis showed broadly consistent pattern, with RHW increases in frequency and duration more pronounced than AHW increases (Extended Data Fig. 3a–c). Between 1981–1990 and 2010–2019, relative increases in RHW frequency, duration and intensity also exceeded those of AHWs (Extended Data Fig. 3d), consistent with the basin-averaged results (Fig. 1). Moreover, increasing trends in RHW frequency, duration and intensity were detected in 65.3%, 92.7% and 93.0% of basins, respectively, all higher than the corresponding proportions for AHWs (48.7%, 72.4% and 76.4%, respectively; Extended Data Fig. 3e,f).

**Extended Data Fig. 4:**
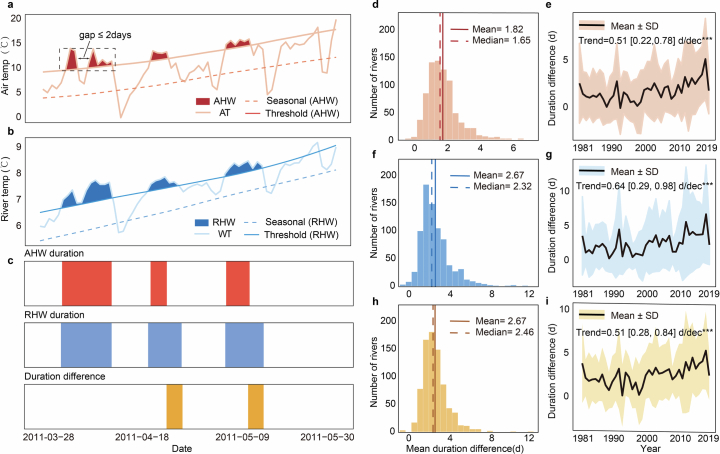
Duration differences between AHWs and RHWs within ARCH. **a–c**, Identified AHWs and RHWs from daily AT and WT, and their duration differences. **d, e**, Distribution and temporal trend of duration differences when AHWs and RHWs use a 3-day threshold. **f, g**, Corresponding results using 5-day threshold. **h, i**, Results under the main definition, with 3-day AHWs and 5-day RHWs. In e, g, and i, lines and shading denote the means ± s.d. across basins (n = 796). Trends and significance were estimated using Theil–Sen analysis. Significance (two-sided test): *P < *0.05*, **P < *0.01*, ***P < *0.001*.

A quantitative analysis reveals that the RHWs last longer than AHWs in 99.6% of compound events (Extended Data Fig. 4a–c). Specifically, the average duration difference is 2.67 days (median: 2.46 days), with the gap exceeding 12 days in extreme cases (Extended Data Fig. 4h). Crucially, this duration gap has also widened over time, increasing by 0.51 days per decade from 1981 to 2019 (Extended Data Fig. 4i). These findings are robust across various heatwave definitions (Extended Data Fig. 4d–g), indicating different thermal inertia and responses of AHWs and RHWs to climate change.

**Fig. 2:**
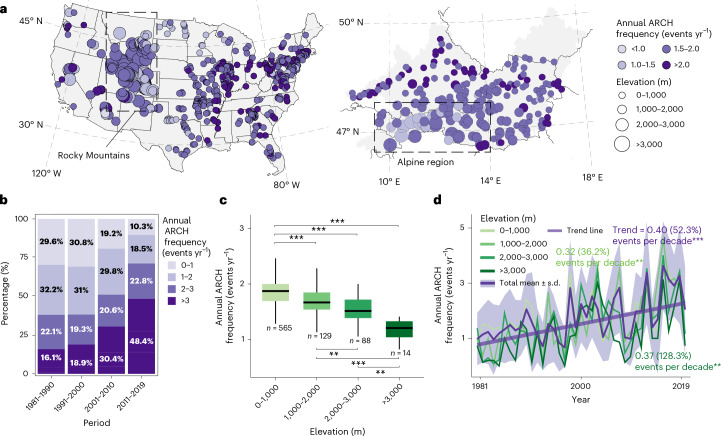
Spatiotemporal distribution of ARCH frequency. **a**, Mean annual ARCH frequency. Dashed boxes mark the Rocky Mountains (mean terrain elevation: 2,037 m) and the Alpine region (2,484 m). **b**, River site proportions in four frequency classes by period. **c**, ARCH frequency by elevation. Boxplots indicate the median, interquartile range (IQR) and 1.5 × IQR whiskers. Groups were compared using two-sided Dunn’s post-hoc test with Benjamini–Hochberg correction. **d**, Annual trends and significance from Theil–Sen analysis; the solid purple line shows the fitted trend, and the shaded area indicates ± s.d. Significance (two-sided test): *P < 0.05, **P < 0.01, ***P < 0.001. Maps in **a** generated with US boundaries data from the US Census Bureau and Central European country boundaries data from Eurostat/GISCO Countries 2020, EuroGeographics.

**Extended Data Fig. 5:**
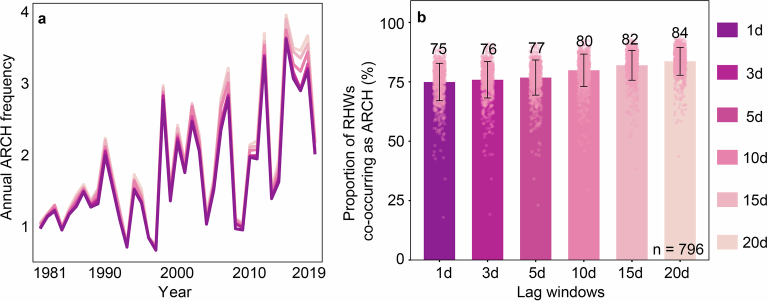
Sensitivity analysis of ARCH identification to lag windows. **a**, Annual ARCH frequency from 1981 to 2019 under six lag-window thresholds of 1, 3, 5, 10, 15 and 20 days. Darker to lighter purple lines denote smaller to larger thresholds. **b**, Proportion of RHWs classified as ARCH under each lag-window threshold across 796 basins (n). Bar labels indicate mean percentages, and error bars show inter-basin standard deviation (n = 796). The results show that ARCH frequency is weakly sensitive to the choice of lag windows.

**Extended Data Fig. 6:**
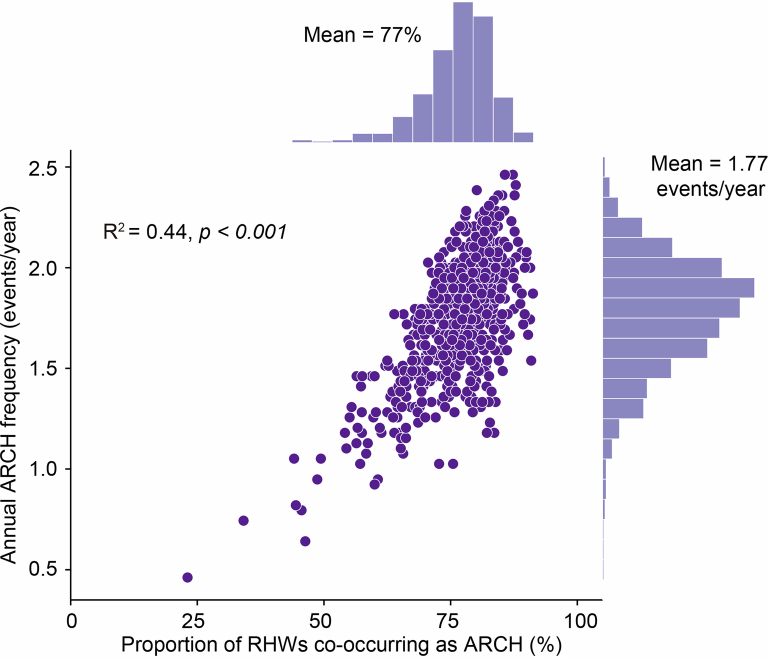
Relationship between ARCH frequency and RHW compound proportion. Across 796 river basins, annual ARCH frequency was positively correlated with the proportion of RHWs occurring as ARCH, with a mean ARCH frequency of 1.77 events per year and a mean RHW compound proportion of 77%. Statistical significance was assessed using a two-sided Pearson correlation test, *P* < *0.001*.

We define ARCHs as events where AHWs and RHWs co-occur within the same basin and during a compound spatiotemporal window spanning from 5 days before the onset of each RHW to its end (see Methods for details). Sensitivity tests using alternative windows of 1, 3, 5, 10, 15 and 20 days confirmed the robustness of ARCH frequency (Extended Data Fig. 5). ARCH events were widespread across the 796 river basins in the USA and CE (Fig. 2a), with an average of 1.77 events occurring per year. Between 1981 and 2019, 77% of all RHWs occurred in the form of ARCHs (Extended Data Fig. 6). Basins with more frequent ARCHs also showed a higher fraction of RHWs occurring as compound events (Extended Data Fig. 6). A temporal trend shows that high-frequency ARCH events expanded substantially over time, and by the 2011–2019 period, 48.4% of river basins experienced at least three ARCH events per year (Fig. 2b). Long-term trend analysis shows that the ARCH frequency increased from 0.76 to about 2.3 events per year since the 1980s (+0.40 events per decade; Fig. 2d), effectively tripling over the past four decades.

ARCH frequency also varied substantially across space, with lower occurrence in high-elevation basins. Specifically, rivers in the Rocky Mountains and the Alps experienced fewer ARCH events than lowland rivers (Fig. 2a), exhibiting a clear decline along an elevation gradient (Fig. 2c). It is noteworthy that although high-elevation river basins (>3,000 m) have a lower average annual ARCH frequency, they exhibit a significantly stronger growth trend (*P* < 0.01; Fig. 2d). Specifically, high-elevation regions (>3,000 m) showed greater increases in ARCH frequency compared with low-elevation regions (<1,000 m), both in absolute (0.37 events per decade) and percentage (128% per decade) terms. A similar pattern is observed in RHWs within the Rocky Mountains and the Alps (Fig. 1a–b and Extended Data Fig. 3a,b). Together, these consistent patterns suggest that mountainous rivers are emerging as fast-warming hotspots of compound heatwave risk, probably driven by elevation-dependent warming that causes high-altitude systems to warm faster than those at lower elevations^27,28^.

## Climate, topographic and hydrological controls

**Fig. 3:**
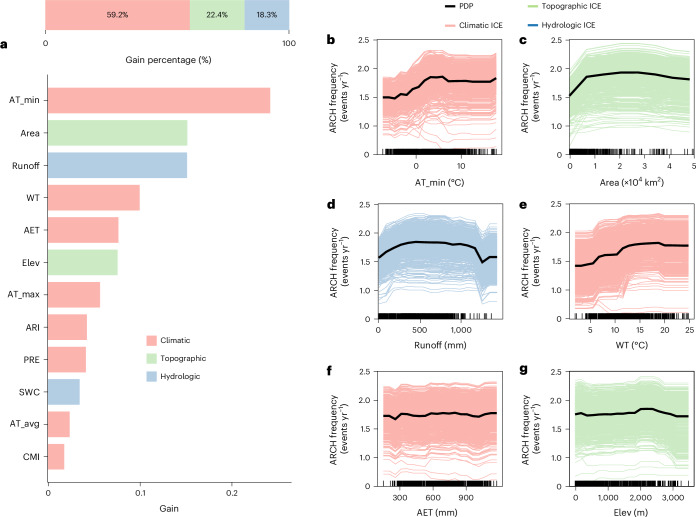
Important drivers of ARCH frequency. **a**, Gain importance of 12 predictors, including AT_min (°C), river area (km^2^), land surface runoff (mm), WT (°C), actual evapotranspiration (AET; mm), elevation (Elev; m), AT_max and AT_avg (°C), aridity index (ARI), precipitation (PRE; mm), soil water content (SWC; %) and climate moisture index (CMI). Predictors are grouped into climatic, topographic and hydrological categories. **b–g**, Individual conditional expectation (ICE) curves show site-level responses; partial dependence plots (PDPs) show the mean response across ICE curves and indicate marginal effects of the six leading predictors: AT_min (**b**), river area (**c**), land surface runoff (**d**), WT (**e**), AET (**f**) and elevation (**g**).

**Extended Data Fig. 7:**
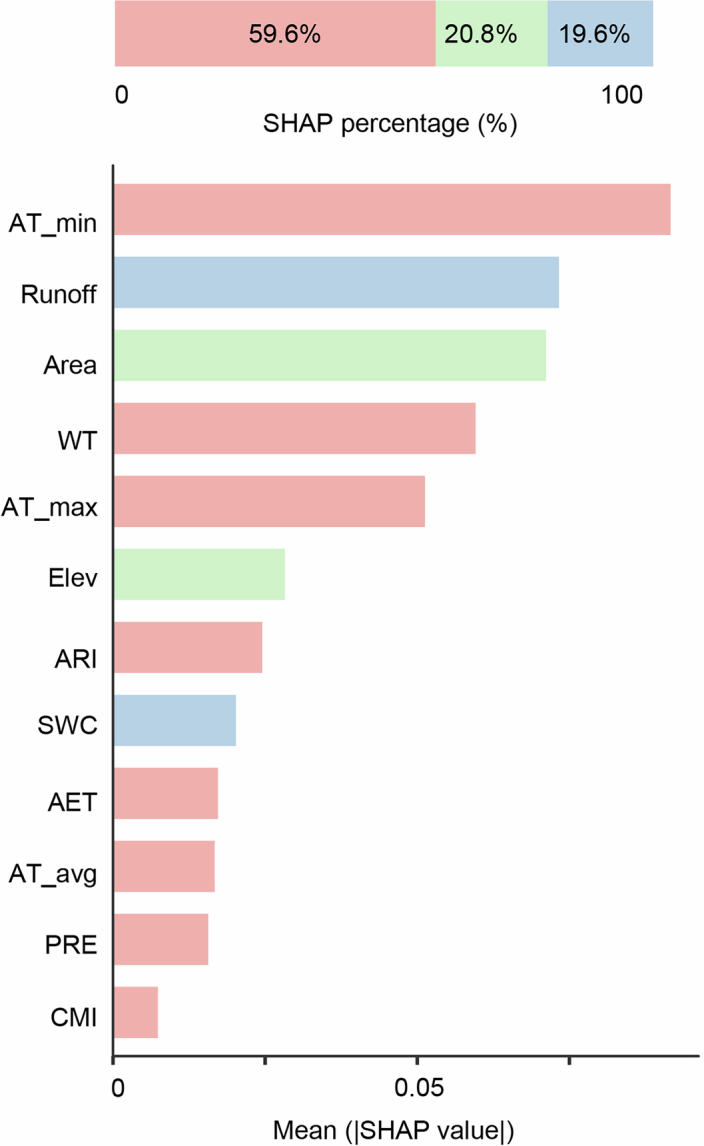
SHAP-based importance of predictors for ARCH frequency. Bar colours indicate climatic, geographic and hydrologic predictor groups. The upper stacked bar summarizes the category-level contribution of each group.

The rapid expansion of ARCHs across basins raises the question of which watershed conditions regulate their occurrence. We identified 12 key attributes associated with the annual frequency of ARCHs and quantified their relative importance using the eXtreme Gradient Boosting (XGBoost) gain scores (Fig. 3a). Among these, climatic factors (primarily air temperature (AT), water temperature (WT), evapotranspiration and precipitation) contributed 59.2% to model performance. In comparison, topographic factors (such as river area and elevation) and hydrological factors (including surface runoff and soil water content) contributed 22.4% and 18.3%, respectively. A supplementary analysis using Shapley additive explanations (SHAP) yielded broadly consistent patterns in both the dominant predictors and the category-level contributions, supporting the robustness of these attribution results (Extended Data Fig. 7).

Minimum AT (AT_min) was the single most important predictor of ARCH frequency, exceeding the maximum and average AT (that is, AT_max and AT_avg; Fig. 3a). ARCH frequency increased markedly with rising AT_min (Fig. 3b), suggesting that nighttime warming promotes thermal accumulation and delays river cooling. This is consistent with previous evidence that sustained high river temperatures are often more strongly associated with elevated nighttime AT than with daytime extremes^22^. Beyond ambient AT, ARCH frequency is sensitive to the river’s thermal state (WT), with the most rapid increases in frequency occurring within the 0–15 °C range (Fig. 3e).

Topographic and hydrological factors also modulate ARCH frequency. For example, ARCH frequency increases rapidly with catchment area up to approximately 10,000 km^2^, after which the effect levels off (Fig. 3c). Elevation has a similarly nonlinear influence (Fig. 3g), with ARCH frequency increasing slightly at mid-elevation ranges of 1,000–2,000 m but declining sharply above 2,000 m. Surface runoff also exhibited a threshold response: ARCH frequency rises with runoff up to approximately 500 mm, after which it gradually declines (Fig. 3d).

## Water quality impacts and future risks

**Fig. 4:**
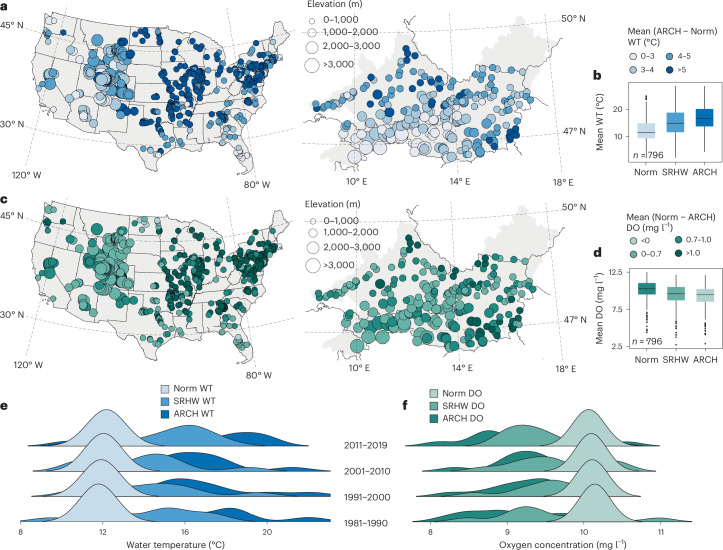
River WT and DO under heatwave conditions. **a**, WT increase under ARCH relative to Norm (calculated as ARCH minus Norm); colour indicates magnitude and symbol size indicates elevation. **b**, Mean WT under Norm, SRHW and ARCH across 796 sites (*n*). **c**, DO decline under ARCH relative to Norm. **d**, Mean DO under the three conditions across 796 sites (*n*). In **b**,**d**, boxplots indicate the median, IQR and 1.5 × IQR whiskers. **e**, Ridgeline distributions of annual mean WT across periods and heatwave conditions. **f**, Corresponding DO distributions. Maps in **a** and **c** generated with US boundaries data from the US Census Bureau and Central European country boundaries data from Eurostat/GISCO Countries 2020, EuroGeographics.

**Extended Data Fig. 8:**
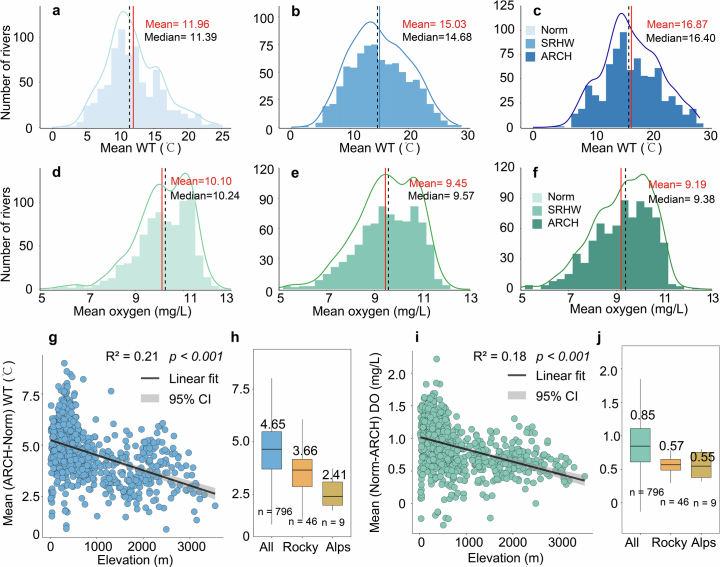
WT and DO under heatwave conditions and elevation-dependent ARCH responses. **a–c**, Basin-level distributions of mean WT under Norm, SRHW and ARCH conditions. **d–f**, Corresponding distributions of mean DO. Solid/dashed vertical lines show means/medians. **g**, Elevation dependence of ARCH-induced WT changes; **h**, WT comparisons for Rocky Mountain basins and high-elevation basins in the Alps above 2100 m. **i**, Elevation dependence of ARCH-induced DO changes; **j**, regional DO comparisons. In **g** and **i**, lines show linear fits and shading denotes 95% confidence intervals (CI). In **h** and **j**, boxplots indicate the median, interquartile range, and 1.5×IQR whiskers.

Dissolved oxygen (DO) is a key indicator of water quality and aquatic ecosystem health^29^. To assess whether compound heatwaves impose stronger water quality stress than isolated RHWs, we examined WT and DO changes under three conditions (Fig. 4): background (Norm, periods without RHWs), single RHW (SRHW, RHWs occurring independently of AHWs) and ARCHs (RHWs compounded with AHWs). ARCH exerts the strongest thermal and oxygen stress among the three conditions studied (Fig. 4b,d). Across the 796 rivers, mean WT under ARCHs reached 16.9 °C, compared with 12.0 °C under Norm conditions and 15.0 °C during SRHW (Extended Data Fig. 8a–c). Relative to Norm conditions, WT increased by approximately 41% during ARCHs and 25% during SRHW, corresponding to an additional 16% increase under compound events. Concurrently, mean DO concentrations reached minimum levels under ARCHs (9.2 mg l^−1^), compared with 10.1 mg l^−1^ under Norm and 9.5 mg l^−1^ during SRHW periods (Extended Data Fig. 8d–f). This corresponds to a 9.8% DO decline relative to non-RHW conditions and a further 2.9% decline compared to SRHWs. The larger relative increase in WT (41%) indicates a tight coupling between atmospheric and water temperature, while the smaller decline in DO (9.8%) suggests additional controls beyond temperature forcing^30^, such as light, flow and anthropogenic influences.

Across 1981–1990, 1991–2000, 2001–2010 and 2011–2019, the most pronounced warming and deoxygenation effects occurred in the most recent decade (Fig. 4e,f). Together, these results show that ARCH events cause more severe water quality deterioration than isolated RHWs, with their impact becoming increasingly pronounced over time. ARCH impacts also varied systematically with elevation. Both ARCH-induced WT increases and DO declines exhibited modest but significant negative relationships with elevation (*P* < 0.001; Extended Data Fig. 8g,i), indicating stronger compound stress in lowland rivers. Specifically, ARCH-induced changes were smaller in the Rocky Mountains (+3.66 °C; −0.57 mg l^−1^) and the Alps (+2.41 °C; −0.55 mg l^−1^) compared with the average (WT: +4.65 °C; DO: −0.85 mg l^−1^; Extended Data Fig. 8h,j). However, the rapid increase of ARCH frequency suggests that mountain rivers may face growing risks under continued elevation-dependent warming.

**Fig. 5:**
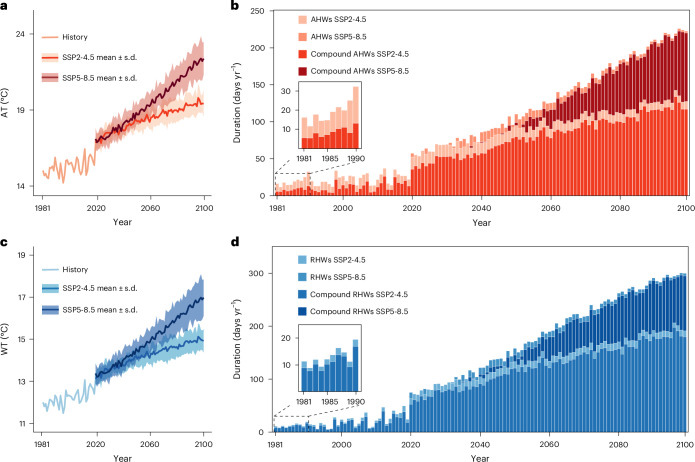
Future projections of heatwaves. **a**, Annual maximum AT under historical and future scenarios. **b**, Annual total duration of AHWs under SSP2-4.5 and SSP5-8.5, with darker segments showing the portion occurring under compound conditions. **c**, Annual maximum WT under historical and future scenarios. **d**, Annual total duration of RHWs under the two scenarios, with darker segments showing the portion occurring under compound conditions. In **a** and **c**, lines and shading denote the multi-model means ± s.d, respectively. Insets in **b** and **d** zoom in on the early historical period, 1981–1990.

Future thermal risks are assessed under the intermediate Shared Socioeconomic Pathway (SSP2-4.5) and the high-emissions pathway (SSP5-8.5). Annual mean AT and WT are projected to increase substantially, accompanied by marked increases in the annual duration of AHWs and RHWs (Fig. 5). Under SSP5-8.5, AHW duration is projected to lengthen by 1.64 days per year from 2020 to 2100, while RHW duration is expected to increase even more rapidly, at a rate of 2.45 days per year. The proportions of heatwave duration occurring under compound conditions also increase under both scenarios, with stronger changes under the high emission scenario (Fig. 5b–d). By 2091–2100, the proportion of AHW days coinciding with RHWs is projected to rise from 43.7% (in 1981–1990) to 98.5%. Similarly, the proportion of RHW days occurring as ARCH events is projected to increase from 84.5% to 98.5%. These findings indicate that AHWs and RHWs will become increasingly synchronized under continued warming, making ARCH events the dominant form of riverine thermal stress by the end of the century.

**Extended Data Fig. 9:**
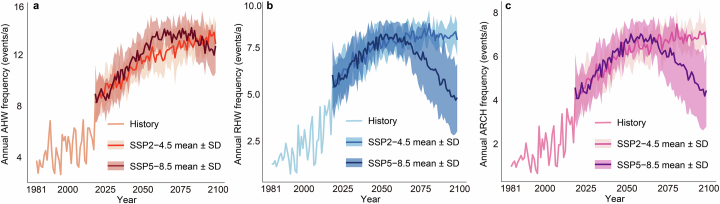
Projected frequencies of AHWs, RHWs and ARCH. **a**, Annual AHW frequency. **b**, Annual RHW frequency. **c**, Annual ARCH frequency. Historical estimates cover 1981–2019, and future projections cover 2020–2100 under SSP2-4.5 and SSP5-8.5. Solid lines show historical estimates or multi-model mean projections, and shaded bands show inter-model standard deviation. Red, blue and purple denote AHWs, RHWs and ARCH, respectively; lighter and darker tones indicate SSP2-4.5 and SSP5-8.5.

We further projected changes in AHW, RHW and ARCH frequency. Under SSP2-4.5, all three event types increase steadily throughout the twenty-first century. Under the SSP5-8.5 scenario, however, AHW and RHW frequencies are projected to decline in the latter half of the century. RHW frequency is expected to peak around mid-century, while AHW frequency shows a clear downward trend after approximately 2075 (Extended Data Fig. 9). This suggests a potential shift from frequent, short-duration events towards fewer but more persistent heatwaves in river systems.

## Discussion

### Rapid intensification of RHWs

Across 796 basins in the USA and CE, RHW duration and intensity increased by 5.92 days per decade and 1.54 °C per decade, respectively, during 1981–2019 (Fig. 1). These rates exceed those reported for lake heatwaves (2.1 ± 0.1 days per decade and 0.4 ± 0.02 °C per decade since 1980; linear trend estimate ± s.e.)^31^ and marine heatwaves (~1.3 days per decade and ~0.085 °C per decade since the early 1980s)^26^. Although such cross-system comparisons are inherently subject to discrepancies in spatiotemporal domains, baseline periods, intensity metrics and event-identification methods, this divergence probably reflects fundamental differences in thermal buffering: rivers are shallow, low-storage systems with strong surface exchange^23^, whereas lakes and oceans are moderated by deeper mixed layers, stratification and circulation^31,32^. Rivers therefore respond more acutely to atmospheric forcing, underscoring RHWs as a distinct and rapidly escalating climate hazard^33^.

### High prevalence of ARCHs

We found strong synchronization between atmospheric and riverine thermal extremes, with 75–84% of RHWs co-occurring with AHWs, far exceeding rates reported in estuaries (36%)^34^ and marine systems (53%)^35^. A recent study of 265 lakes similarly showed that atmosphere–lake coupled heatwaves intensify event severity when they co-occur^36^. Such high ARCH prevalence is attributable to the rapid thermal response of rivers, where high surface-area-to-volume ratios and turbulent mixing facilitate the efficient transmission of atmospheric anomalies (for example, solar radiation, near-surface AT) to the water column^23^. Conversely, we observe that ~20% of RHWs occur in the absence of concurrent atmospheric extremes (Extended Data Fig. 5). This decoupling probably reflects the combined influence of natural and anthropogenic controls. Natural processes, including groundwater–surface water interactions^37^, riparian shading^38^ and water-column thermal inertia^39^, can modulate the coupling between AT and WT. Human activities can further reshape these thermal regimes. For example, reservoir operations may buffer downstream temperatures by up to 6.6 °C (ref. ^40^), whereas thermal effluents can induce localized river warming in the absence of atmospheric extremes^41^.

### Distinct drivers and coupling mechanisms

Our analysis attributes ~60% of variation in ARCH frequency to climate change, with AT_min emerging as the dominant driver (Fig. 3). This dependency aligns with established evidence that rising minimum temperature promotes river heat extremes^42^. However, ARCH formation depends not only on atmospheric heating, but also on the efficiency of air–water energy exchange. Consequently, variables related to latent and sensible heat fluxes, including evapotranspiration and moisture conditions such as aridity index, also ranked highly (Fig. 3). More broadly, while atmospheric warming affects all aquatic systems, their coupling pathways differ^43,44^. Rivers are open, advection-dominated systems, where WT responds rapidly to short-term anomalies in radiation and near-surface AT^23^. This response is modulated by local watershed conditions, but remains primarily atmospherically driven. In contrast, lakes are strongly buffered by thermal stratification, with deep hypolimnetic waters largely insulated from short-lived AHWs that affect the surface^31^. Oceans operate at much larger scales governed by mixed-layer dynamics and global circulation; in some regions, marine heat anomalies can feed back onto atmospheric circulation and amplify coastal terrestrial heatwave exposure^45^.

### Compound risks of water quality

Our results highlight that ARCH exerts a synergistic amplification of water quality deterioration. Compared with isolated RHWs, ARCH events drive an additional 16% rise in WT and a further 2.9% decline in DO (Fig. 4). These findings place ARCHs within a broader class of compound aquatic extremes, in which co-occurring stressors impose nonlinear ecological risks. Similar synergistic effects have been reported in other aquatic systems. In lakes, heatwaves compounded by atmospheric stilling can intensify stratification and oxygen depletion^46^, whereas in oceans, marine heatwaves overlapping with acidification can produce impacts far greater than the sum of their individual effects^47^. These findings suggest that compound extremes are becoming an increasingly important driver of water quality deterioration across aquatic systems. Although long-term data availability and model capacity currently limit our assessment to the USA and CE, the continued expansion of global monitoring networks could refine ARCH risk assessments in future studies.

### Tipping points in river thermal dynamics

In the Earth system, tipping points refer to critical thresholds where global warming triggers catastrophic and often irreversible changes to climate and hydrological systems^48^. Scientists warn that several tipping points may already be underway as global temperatures approach 1.5 °C above pre-industrial levels, including melting ice sheets, coral reef collapse and Amazon rainforest dieback^49,50^. Our findings suggest that riverine thermal systems may be approaching a similar transition under continued warming. The projected frequencies of AHWs, RHWs and their compound ARCH events show clear tipping-point behaviour (Extended Data Fig. 9) under the SSP5-8.5 scenario, with heatwaves becoming less frequent but increasingly prolonged and persistent. Similar patterns have also been reported in marine and lake systems^16,17^. Together, these findings suggest that continued warming may drive aquatic systems towards a regime shift, from episodic extremes to chronic thermal stress.

## Methods

### Data sources and processing

We assembled a basin-scale dataset for 796 river basins^30^ across the USA and CE to reconstruct daily WT and DO and to characterize AHWs, RHWs and ARCHs during 1981–2019. The dataset included irregularly sampled WT and DO observations, daily hydrometeorological forcings, and static basin attributes. Daily forcings include AT, precipitation, discharge, snow water equivalent, surface pressure and wind speed. Static basin attributes described geography, topography, long-term climate, hydrological regime, land cover and soil properties. Together, these dynamic and static predictors were used to represent temporal forcing and spatial heterogeneity in WT and DO dynamics.

For the USA, WT and DO observations were obtained from the USGS National Water Information System using the dataRetrieval R package (https://github.com/USGS-R/dataRetrieval). Hydrometeorological forcings were derived from the datasets of NLDAS-2 (https://ldas.gsfc.nasa.gov/nldas/v2/forcing) and DAYMET (https://daymet.ornl.gov/). Specifically, NLDAS-2 provided daily air temperature, wind speed and surface pressure, whereas DAYMET supplied daily maximum and minimum AT, precipitation and snow water equivalent. These forcing variables were aggregated to the basin scale using Google Earth Engine (https://earthengine.google.com/). For CE, WT and DO, observations were collected from national and regional monitoring agencies, and the corresponding hydrometeorological forcings were obtained from the LamaH-CE dataset^51^. All daily forcing variables covered the period from 1 January 1981 to 31 December 2019. Basin attributes for the USA and CE were derived from GAGES-II^52^ and LamaH-CE, respectively. These observations, forcing and attribute datasets were harmonized at the basin scale to support reconstruction of continuous WT and DO records and subsequent ARCH analysis.

### Model evaluation and future projections

We used a trained cross-continental long short-term memory (LSTM) model^30,53,54^ to reconstruct daily WT and DO for 796 rivers across the USA and CE during the period of 1981–2019. We applied a flexible data-splitting strategy by partitioning each basin’s time series into 75% for training and 25% for testing to ensure sufficient coverage for model learning. Four statistical metrics were used for model evaluation, including percent bias (Pbias), Nash–Sutcliffe efficiency (NSE), root mean square error (RMSE) and Pearson’s correlation coefficient (Pcorr; Extended Data Fig. 1). An NSE score <0 indicates unacceptable performance where model prediction is worse than the observational mean, while an NSE ≥0.5 is considered as good performance for the LSTM model at the daily scale^55^. Across all river basins, the LSTM model achieved a median testing NSE of 0.72 for DO, and showed a more robust performance for WT, with the 25th, 50th and 75th percentiles of testing NSE being 0.90, 0.95 and 0.97, respectively (Extended Data Fig. 1). To assess the model’s performance in predicting river WT across different levels of accuracy, we selected four representative basins with testing NSE values corresponding to the 25th, 50th, 75th and 100th percentiles. For each site, we generated daily time series plots of simulated and observed WT, along with zoomed-in views during periods of extreme temperature (Extended Data Fig. 2).

Ten Coupled Model Intercomparison Project phase 6 (CMIP6) models were subsequently selected from the NEX-GDDP-CMIP6 dataset^56^ to project future AT and WT under the SSP2-4.5 and SSP5-8.5 scenarios. To ensure a comprehensive representation of global model development institutions relevant to our study regions (the USA and CE), we chose seven models from North American and European centres and three from Asia^30^. The selected models include GFDL-ESM4, GISS-E2-1-G and CanESM5 (North America); EC-Earth3, MPI-ESM1-2-HR, CMCC-ESM2 and IPSL-CM6A-LR (Europe); and BCC-CSM2-MR, MIROC6 and MIROC-ES2L models (Asia). The projected variables were daily maximum AT and daily river WT. All processing was similarly performed at the basin scale to drive daily model projections from 2020 to 2100 under the SSP2-4.5 and SSP5-8.5 scenarios.

### Definition and identification of AHWs and RHWs

To ensure consistent identification of extreme heat events across different domains^44,57^, we used definitions that account for differences in heat capacity between the atmosphere and rivers and in the thermal tolerances of terrestrial and aquatic organisms^2,58^. Following established methods, RHWs and AHWs were defined as periods of at least 5 and 3 consecutive days, respectively, during which daily river WT and daily maximum AT exceeded the 90th percentile of the local seasonal climatology^22,59^. AHWs and RHWs were identified using the heatwaveR package (https://github.com/robwschlegel/heatwaveR), and consecutive events separated by less than 3 days were considered a single event. The climatological baseline and 90th percentile threshold were calculated for each calendar day using an 11-day window centred on that date across all years in the 1981–2019 reference period, and the resulting time series was smoothed by a 31-day moving average^26,32^. The 11-day window is widely adopted in heatwave studies^17^ to ensure robust sample size for percentile estimation while preserving the seasonal evolution of the climatology. This seasonally varying threshold enables year-round detection of locally rare thermal events, rather than relying on fixed or absolute temperature thresholds that may not be transferable across climatic regions. In line with established lake and marine heatwave studies, this percentile framework also enables comparisons across different ecosystem types. Because the thresholds are based on a fixed historical climatology, trends in frequency should be interpreted as changes relative to the 1981–2019 baseline and may reflect both increased short-term heat extremes and shifts in the mean thermal regime under warming.

To compare RHWs and AHWs, we evaluated three key metrics: frequency (the number of heatwave events per unit of time), intensity (the average temperature relative to the climatological mean during an event) and duration (the number of days between the start and end of an event). In cases where a single heatwave spanned 31 December and 1 January, the event was partitioned and attributed to its respective calendar year. To ensure the robustness of our results, we applied the Theil–Sen analysis to quantify both absolute and percentage changes in the time series, thereby minimizing the influence of outliers on the trend analysis.

### ARCHs

To identify ARCHs, we adapted an event-synchronization approach commonly used to link rainfall and runoff events^60,61^. This approach accounts for the potential lag between atmospheric heat extremes and river thermal responses. For each RHW, we defined a compound window extending from *n* days before the onset of the RHW to the end of the RHW, where *n* is a user-defined lag parameter. In the main analysis, we set *n* to 5 days. An RHW was classified as an ARCH if at least one AHW occurred within this compound window, that is, from 5 days before the RHW onset to the end of the RHW. To assess sensitivity to the choice of lag window, we recalculated ARCH frequency and the proportion of RHWs classified as ARCHs across all 796 basins using alternative values of *n* = 1, 3, 5, 10, 15 and 20 days. These sensitivity results are shown in Extended Data Fig. 5.

Additionally, we quantified differences in event duration between RHWs and AHWs under compound ARCH conditions (Extended Data Fig. 4). Because an RHW can overlap with multiple AHWs, we first summed the durations of all AHWs detected within the compound window to estimate the total atmospheric heatwave duration associated with each ARCH. The duration difference was then calculated as the RHW duration minus the summed AHW duration within the corresponding compound window. For analyses of interannual variability, each ARCH was assigned to the year in which the RHW began. We then calculated the annual mean duration difference across all ARCHs in each basin and year.

### Identifying and evaluating influential drivers

To identify the key factors influencing the annual ARCH frequency, we applied the XGBoost model for feature importance analysis. XGBoost is a machine learning algorithm based on gradient boosting decision trees^62^, which has been widely used in environmental and climate studies due to its strong ability to capture nonlinear relationships^63^. In this study, we compiled 197 attribute variables for 796 river basins from the HydroATLAS^64^ database, including variables related to basin topography, climate, land cover, soil, geology and anthropogenic influence. After manually removing variables representing monthly data (for example, tmp_dc_s07, aet_mm_s04), we employed the VSURF^65^ package to select 12 variables that best explained the target variable. The VSURF algorithm incrementally introduces variables and monitors changes in the out-of-bag error, stopping the selection process once additional variables no longer improve model performance. Grid search was subsequently used to optimize the XGBoost model with final hyperparameters as max_depth = 4, eta = 0.2, subsample = 0.7, colsample_bytree = 0.8 and nrounds = 68. The influential indexes were ranked based on gain, the average improvement in model performance when including the index. The final model achieved an *R*^2^ of 0.74 for the testing set, indicating robust performance. Individual conditional expectation (ICE) plots and partial dependence plots (PDPs) were used to visualize the dependence of annual ARCH frequency on influential drivers^66^. ICE plots illustrate how the annual ARCH frequency at individual sites changes as the value of a specific predictor varies, while all other predictors are held constant^67^. The PDP is the average of ICE plots for individual sites and represents the average response of annual ARCH frequency to variations of an individual driver.

## Online content

Any methods, additional references, Nature Portfolio reporting summaries, source data, extended data, supplementary information, acknowledgements, peer review information; details of author contributions and competing interests; and statements of data and code availability are available at 10.1038/s41561-026-02040-y.

## Supplementary information

Peer Review file

## Data Availability

US datasets: WT and DO observations were accessed from the USGS National Water Information System (NWIS) at https://waterdata.usgs.gov/nwis. Meteorological forcings were obtained from NLDAS-2 at https://ldas.gsfc.nasa.gov/nldas/v2/forcing and DAYMET at https://daymet.ornl.gov. Basin attributes were derived from GAGES-II at https://water.usgs.gov/GIS/metadata/usgswrd/XML/gagesII_Sept2011.xml. Central Europe datasets: meteorological forcings and basin attributes were obtained from LamaH-CE available at 10.5194/essd-13-4529-2021 (ref. ^51^). WT and DO observations for Central Europe were obtained from national and regional data providers, including the Swiss Federal Institute of Aquatic Science and Technology and the Federal Office for the Environment (10.25678/0004AV), the Austrian Federal Ministry of Agriculture, Regions and Tourism (https://wasser.umweltbundesamt.at/h2odb/fivestep/abfrageQdPublic.xhtml), the State Agency for the Environment Baden-Württemberg (https://udo.lubw.baden-wuerttemberg.de/public/index.xhtml) and the Bavarian State Office for the Environment (https://www.gkd.bayern.de/en/rivers/chemistry). Supporting data for this study are available at https://github.com/YuZhouWater/atmospheric-riverine-compound-heatwaves/tree/main/data. Future climate projections from NEX-GDDP-CMIP6 are available at 10.7917/OFSG3345.

## Extended data

**Extended Data Table 1:** Trends in AHW and RHW frequency, duration, and intensity under different duration thresholds

Heatwave_types	Key_metrics	Absolute_change_x10	Slope_95_interval	Relative_change_percent		Statistical_significance
AHWs (5d)	Frequency	0.25	[0, 0.36]	35.6		*P* = 0.053
AHWs (5d)	Duration	1.49	[0.01, 2.8]	40.1		*P* = 0.050
AHWs (5d)	Intensity	1.56	[−0.08, 2.64]	30.9		*P* = 0.060
RHWs (3d)	Frequency	0.98	[0.62, 1.27]	84.2		*P* < 0.001
RHWs (3d)	Duration	7.72	[4.89, 10.89]	121		*P* < 0.001
RHWs (3d)	Intensity	2.72	[1.58, 4.18]	72.6		*P* < 0.001

AHWs (RHWs) are identified by daily maximum temperatures (daily river water temperatures) being above the 90th percentile threshold of local and seasonal variability for at least five days (three days). Trends and significance (two-sided test) were estimated using Theil–Sen analysis.

## Code availability

Code used to analyse data and develop the figures is available via GitHub at https://github.com/YuZhouWater/atmospheric-riverine-compound-heatwaves.
