# Supplementary material for: Threefold increase in atmospheric–riverine compound heatwaves under climate change
Source: Nat Geosci. 2026 Jul 30;19(8):906–13. doi: 10.1038/s41561-026-02040-y (PMC13447100; doi:10.1038/s41561-026-02040-y)
Supplement: Supplementary file 1 [file 41561_2026_2040_MOESM1_ESM.pdf]

nature portfolio

Peer Review File

Threefold increase in atmospheric-riverine compound heatwaves under climate change

Corresponding Author: Dr R. Iestyn Woolway

Version 0:

Reviewer comments:

Reviewer #1

(Remarks to the Author)
Major Comments
This manuscript introduces a novel concept of atmospheric–riverine compound heatwaves (ARCH) and provides a broad assessment of trends, drivers, and water-quality impacts across 796 basins in the US and Europe. Given the growing recognition of riverine thermal extremes and their emerging risks, shifting the research focus toward the synergistic effects of compound heatwaves is timely, relevant, and essential for improving water risk assessments. The paper is generally well structured and the analyses are extensive, spanning diverse hydro-climatic conditions across continents. Two findings are particularly intriguing and could make the study a valuable contribution with further development: 1) the elevation-dependent of ARCH risk, whereby high-elevation mountain rivers (>3000 m) currently exhibit a lower baseline frequency of ARCH events yet show the most rapid intensification; and 2) a projected "tipping-point" transition toward persistence-dominated heat extremes, in which individual events increasingly coalesce into near-continuous, long-duration thermal stress. Therefore, I would recommend a major revision to improve data analysis, refine figure presentation, and strength the relevant discussion. The following points, if addressed, would help the manuscript become a timely and impactful contribution to the emerging field of riverine heatwave research.
First, regarding Figure 1, the reported trends are primarily presented as aggregated regional averages. Given the strong spatial heterogeneity inherent in these hydrological systems across continents, relying solely on large-scale averages can easily mask localized extremes or obscure opposing trends within the same region. To address this, please add a map-based analysis (perhaps complemented by boxplot) to provide the full distribution of basin-level trends. Furthermore, I recommend explicitly reporting the fraction or percentage of individual basins exhibiting statistically significant increases versus decreases. This addition would allow readers to assess the spatial consistency of the historical changes and identify potential hotspots of vulnerability.
Second, the current feature importance analysis is based on XGBoost's built-in gain metric, which measures the average improvement in prediction accuracy contributed by each feature. While this approach is widely used and provides useful insights, it has a few known limitations. Given the growing adoption of SHAP (SHapley Additive exPlanations) in hydrological and climate impact studies, I would be curious to see whether the identified influential drivers remain roughly consistent under a SHAP-based framework. To ensure the robustness of your physical interpretations, please consider adding a supplementary figure with SHAP-based feature importance. This would strengthen the credibility of the driver attribution and make the interpretation more transparent.
The results suggest an elevation-dependent pattern in WT and DO changes. While the analytical framework is conceptually sound, the current presentation remains largely qualitative (e.g., phrases such as "substantial WT increases" and "smaller DO declines") and therefore lacks quantitative rigor. To strengthen this section, please add a more in-depth statistical analysis that explicitly quantifies the elevation dependence. In particular, I would recommend the authors to provide targeted quantification for the U.S. Rocky Mountains and the European Alpine regions—already highlighted in Figure 2—so readers can assess the magnitude and robustness of changes in these key high-elevation hotspots. If necessary, consider using supplementary figures to visualize the elevation-dependent relationships more clearly.
The manuscript identifies a potentially important "tipping-point" transition (Figure 5 and S6): a notable regime shift where projected riverine heatwaves become less frequent but longer-lasting. This pattern may indicate that warming shifts river systems from episodic extremes toward chronic thermal stress, where individual events merge into longer episodes. However, the current manuscript does not sufficiently develop or contextualize this important finding. Please consider adding a dedicated discussion paragraph that 1) frame and discuss the tipping-point phenomenon within the broader context of climate and hydrology science; and 2) places the riverine results alongside the marine and lake heatwave literature. For example, whether similar projections have reported intensifying and lengthening heatwaves, potentially trending toward near-permanent conditions in other systems.

Specific comments

1.There are several grammar errors. For example: Line 43, “Global high temperature records continues …” should be “continue”; Line 64, “RHWs is increasing faster”.
2. In Line 57, there is an extra space before the citations “21, disrupt ecosystem metabolism 22,23, ”.
3. Line 91, Figures 1 and S1 look clear and well presented. In the caption, please specify the “full study period”. Also, I could not find the label “p < 0.001 (***)” in the figures.
4. Figure 2, it may be helpful to add the mean elevations of the Rocky Mountains and the Alps (e.g., in the figure panel or caption) to provide context and help readers interpret the patterns more easily.
5. High-elevation mountains exhibit a clearly lower occurrence yet a higher growth trend of ARCH events (Figure 2). What about RHWs? Could this be related to elevation-dependent warming?
6. Line 248, this paragraph currently lacks a clear rationale for analyzing dissolved oxygen. Consider adding a brief sentence to justify DO as a key indicator of water quality.
7. The manuscript occasionally uses inconsistent terminology for event frequency, such as “counts per year” and “events per year”. Check for consistency.
8. Line 346, Figure S6 shows an intriguing tipping point, where future riverine heatwaves tend to become less frequent but more persistent. Have similar phenomena been observed in other water systems?
9. Line 377, where is the supporting evidence for “~20% of RHWs occur in the absence of concurrent atmospheric extremes”?
10. Line 397, correct the typo “Although this responses is”
11. Line 408, please clarify what “an additional 16%” is relative to.
12. The section of High prevalence of ARCH is interesting and insightful, especially in comparison with estuaries and marine systems. Have lake studies reported comparable ratios for atmospheric-lake compound heatwaves?
13. Before conclusion, it is necessary to add a few sentences outlining the key limitations of the data and the modeling approach.
14. Line 486, check format “25th, 50th, and 75th” with superscripts.
15. Line 508, the threshold definition for daily maximum air temperature is unclear.
16. Line 511, clarify the rationale for using the 11-day window, and provide supporting references (if available)?
17. Line 580: It is not clear how the “197 regional attribute variables” were obtained and processed. Please add a brief description.
18. Line 581-582, clarify what kind of monthly data were removed?
19. For the XGBoost model, consider adding a supplementary table summarizing the tuned hyperparameters.

(Remarks on code availability)

Reviewer #2

(Remarks to the Author)
This manuscript investigates atmospheric–riverine compound heatwaves (ARCH) across 796 river basins in the US and Central Europe. The reported trends, especially the rapid growth of ARCH in high-elevation areas and their disproportionate impact on water quality (warming and deoxygenation), represent a potentially valuable contribution to the field. The topic is timely, relevant to the scope of the journal, and likely to be of interest to a broad readership. The manuscript is generally well organized and technically solid, and I would recommend a major revision to improve the rigor, clarity, and overall presentation of the manuscript.

1. In Fig. 2c, the authors use a density plot to show the distribution of ARCH frequency across different elevation gradients. The overall pattern is generally clear, and it is possible to see that high-elevation mountain rivers above 3000 m tend to have lower ARCH frequency overall. However, I am concerned that the similar color palettes and overlapping curves may reduce the visual clarity of this result. This may also make it more challenging for readers to directly compare differences among elevation groups. The authors may wish to consider replacing this panel with a boxplot and adding significance testing among elevation groups, so that the group-level differences can be presented more clearly.

2. The manuscript reports that, relative to normal conditions, compound heatwaves are associated with an approximately 41.0% increase in WT and a 9.8% decline in DO. While these numbers and figures effectively illustrate the impact of compound events, the pronounced disparity in the magnitude of these responses deserves further elaboration. I recommend that the authors draw more on the literature concerning riverine dissolved oxygen dynamics and their controls, and expand the analysis from the perspective of multiple interacting processes, in order to better explain this asymmetry. Such discussion would strengthen the mechanistic interpretation of the results.

3. The authors use SSP2-4.5 and SSP5-8.5 to project future heatwave changes, which is an appropriate selection of scenarios. However, the manuscript currently provides only limited context regarding the socioeconomic narratives and climate forcing levels represented by these pathways. I therefore recommend that the authors include a concise briefing at the beginning of the relevant section to define these scenarios. Specifically, clarifying that SSP2-4.5 represents a "Middle of the Road" pathway with intermediate forcing, while SSP5-8.5 denotes a high-emission, fossil-fuel-intensive development scenario, would be beneficial.

4. The discussion notes that approximately 20% of RHWs occur independently of AHWs. This is an intriguing result and may suggest a certain degree of decoupling between water and air temperature. The authors currently attribute this pattern primarily to anthropogenic regulation, which is a plausible explanation. However, natural controls should also not be overlooked. Beyond human intervention, the intrinsic thermal buffering capacity of river systems, driven by processes such

as groundwater-surface water interactions (hyporheic exchange), riparian shading, and the thermal inertia of large water bodies, may play a critical role in modulating this decoupling. I therefore encourage the authors to expand this section by incorporating relevant literature on these natural factors, so as to provide a more balanced discussion.

5. Other minor comments regarding language, formatting, and notation are as follows:
(1) Line 43: “Global high temperature records continues to be broken” contains a subject–verb agreement error.
(2) Line 53: This sentence appears to contain a grammatical error.
(3) Line 395: “underling” appears to be a typo and should likely be “underlying”.
(4) The expression used for ARCH frequency is not fully consistent, with “frequency”, “counts”, and “occurrences” used interchangeably.
(5) The caption of Fig. 2 contains interpretive statements that may be more appropriate for the main text rather than the figure legend.
(6) The use of “MWT” and “WT” is not fully consistent; the main text mostly uses WT, whereas Fig. 3 uses MWT.
(7) The legend in Fig. 2b lacks units.
(8) The definition of the shaded areas in Fig. 5 is not consistent across the text and caption. In the main text, the shaded area is described as “se” (Line 323), whereas in the figure it is labeled as “std”. This should be clarified.
(9) The formatting of date ranges is inconsistent, with some instances using an en dash and others a hyphen.
(10) The notation for elevation thresholds is inconsistent, including “>3000 m”, “3000 m+”, and “3000+ m”. A standardized format should be used throughout.
(11) Line 191, the expression “improved model performance by 59.2%” may not be fully accurate. The authors may wish to revise this to “contributed to model performance” or similar wording.
(12) Line 397: “this responses is modulated” contains a grammatical error. Please correct it.
(13) Spacing and notation for numbers, units, and percentages should be checked throughout the manuscript. For example, in Line 1004, “25th, 50th” should be formatted as superscripts.
(14) Reference 43 appears incomplete and lacks full bibliographic information. References 3 and 116 refer to the same paper.
(15) Supplementary Figure S8, it is not clear whether the reported NSE values refer to the training or testing period. This should be clarified.

(Remarks on code availability)
The authors have provided a well-documented code repository that includes a comprehensive README file. The instructions for environment setup and execution are clear, and the code is runnable and able to reproduce the main figures presented in the manuscript.

More broadly, I believe the code will be a valuable resource for the community, particularly for researchers working on riverine heatwaves and related hydroclimatic extremes.

Version 1:

Reviewer comments:

Reviewer #1

(Remarks to the Author)
The revised manuscript, "Threefold increase in atmospheric–riverine compound heatwaves under climate change," addresses an important and timely knowledge gap by investigating the occurrence, trends, drivers, and water-quality consequences of compound atmospheric–riverine heat extremes. The revision responds well to the editor's key concerns by providing stronger evidence for recent changes and underlying mechanisms, and by better articulating how these compound extremes may push river systems toward critical thresholds under future climate warming. These additions further strengthen the manuscript's novelty, mechanistic depth and broader relevance to climate-impact science.

Overall, I have carefully reviewed both the revised manuscript and the detailed response to comments. The authors have done a thorough and meticulous job in addressing the comments raised by the editor and the two reviewers, including those related to the figures, methodology, and discussion. The revised manuscript has therefore been substantially improved, and I am very supportive of its publication in its current form.

(Remarks on code availability)

Reviewer #2

(Remarks to the Author)
The authors have addressed my comments well, I have no other comments

(Remarks on code availability)
The code is great.

Open Access This Peer Review File is licensed under a Creative Commons Attribution 4.0 International License, which permits use, sharing, adaptation, distribution and reproduction in any medium or format, as long as you give appropriate credit to the original author(s) and the source, provide a link to the Creative Commons license, and indicate if changes were made.
In cases where reviewers are anonymous, credit should be given to 'Anonymous Referee' and the source.
The images or other third party material in this Peer Review File are included in the article's Creative Commons license, unless indicated otherwise in a credit line to the material. If material is not included in the article's Creative Commons license and your intended use is not permitted by statutory regulation or exceeds the permitted use, you will need to obtain permission directly from the copyright holder.
To view a copy of this license, visit https://creativecommons.org/licenses/by/4.0/

Response to Review Comments

REVIEWER COMMENTS:

Reviewer #1 (Remarks to the Author):

Major Comments

This manuscript introduces a novel concept of atmospheric–riverine compound heatwaves (ARCH) and provides a broad assessment of trends, drivers, and water-quality impacts across 796 basins in the US and Europe. Given the growing recognition of riverine thermal extremes and their emerging risks, shifting the research focus toward the synergistic effects of compound heatwaves is timely, relevant, and essential for improving water risk assessments. The paper is generally well structured and the analyses are extensive, spanning diverse hydro-climatic conditions across continents. Two findings are particularly intriguing and could make the study a valuable contribution with further development: 1) the elevation-dependent of ARCH risk, whereby high-elevation mountain rivers (>3000 m) currently exhibit a lower baseline frequency of ARCH events yet show the most rapid intensification; and 2) a projected "tipping-point" transition toward persistence-dominated heat extremes, in which individual events increasingly coalesce into near-continuous, long-duration thermal stress. Therefore, I would recommend a major revision to improve data analysis, refine figure presentation, and strength the relevant discussion. The following points, if addressed, would help the manuscript become a timely and impactful contribution to the emerging field of riverine heatwave research.

Response: We sincerely appreciate the reviewer's constructive and insightful comments that have helped improve the manuscript. We are grateful for the reviewer's positive evaluation of the overall scope and potential contribution of this study.

As suggested, we have expanded the analysis of elevation-dependent of ARCH risk, refined Extended Data Fig. 3, 7 and 8, and strengthened the discussion of "tipping-point" (Lines 289-300). All points are well taken and revised accordingly. Please see below detailed responses.

First, regarding Figure 1, the reported trends are primarily presented as aggregated regional averages. Given the strong spatial heterogeneity inherent in these hydrological systems across continents, relying solely on large-scale averages can easily mask localized extremes or obscure opposing trends within the same region. To address this, please add a map-based analysis (perhaps complemented by boxplot) to provide the full distribution of basin-level trends. Furthermore, I recommend explicitly reporting the fraction or percentage of individual basins exhibiting statistically significant increases versus decreases. This addition would allow readers to assess the spatial consistency of the historical changes and identify potential hotspots of vulnerability.

Response: We thank the reviewer for this constructive comment. To address this

concern, we have added a new figure (Extended Data Fig. 3) together with spatial analyses. The results are consistent with the regional average shown in Fig. 1. Revisions and changes are as follows:

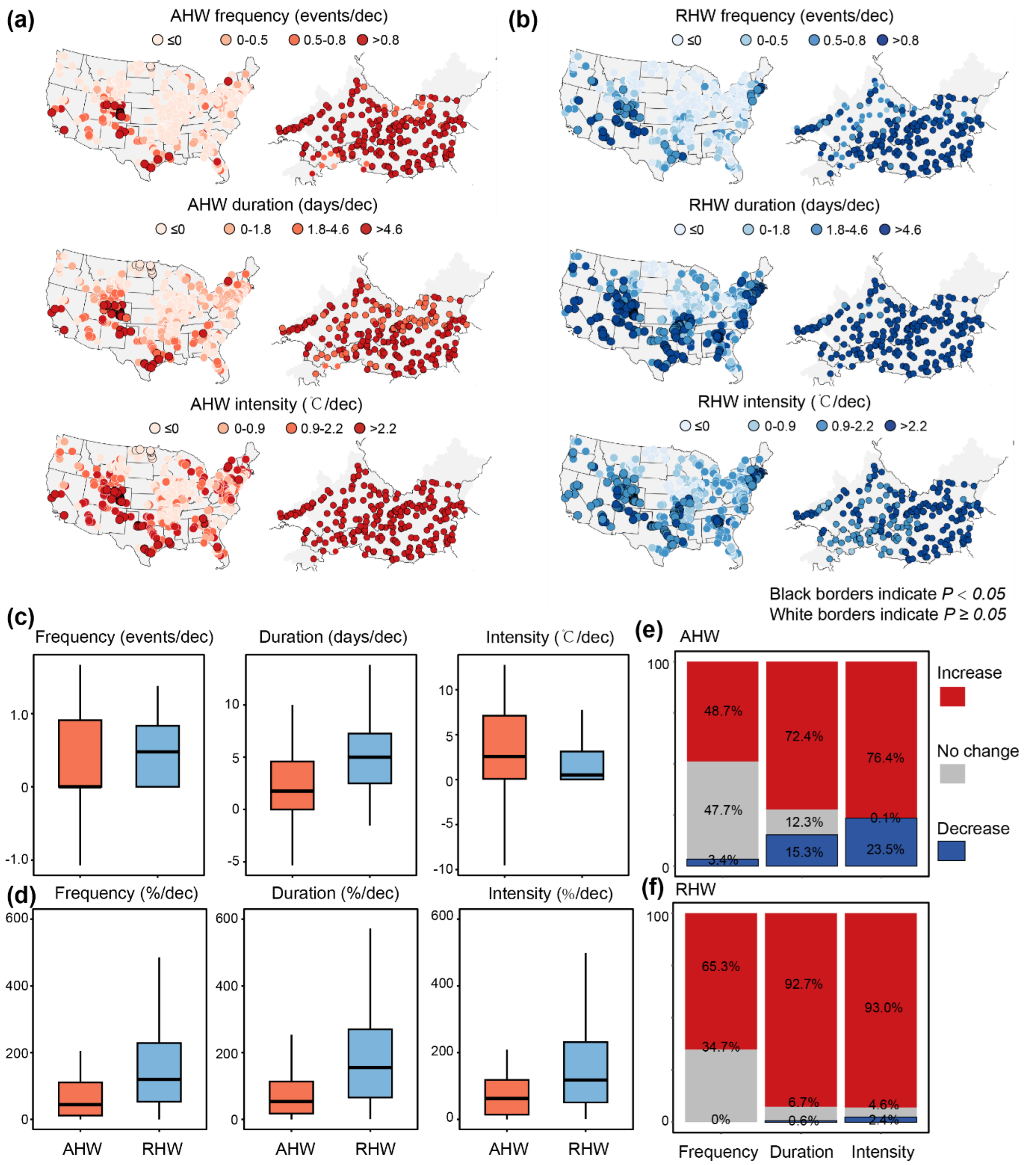

Extended Data Fig. 3 | Basin-level changes in AHW and RHW metrics, 1981–2019. *(a, b) Maps of absolute changes in AHW and RHW frequency, duration and intensity across 796 basins in the US and CE; black and white borders indicate significant and non-significant trends, respectively. (c, d) Basin-level distributions of absolute and percentage changes in frequency, duration and intensity for AHWs and RHWs. (e, f) Fractions of basins showing increasing, decreasing or non-significant trends in AHW and RHW metrics.*

(1) map-based analyses with boxplots (Extended Data Fig. 3a–d) were added to show the full distribution of basin-level trend. The corresponding descriptions have been added as well.

Lines 81-85: "*Spatial analysis showed broadly consistent pattern, with RHW increases in frequency and duration more pronounced than AHW increases (Extended Data Fig. 3a–c). Between 1981–1990 and 2010–2019, relative increases in RHW frequency, duration and intensity also exceeded those of AHWs (Extended Data Fig. 3d), consistent with the basin-averaged results (Fig. 1).*"

(2) the fractions of significant increases and decreases (Extended Data Fig. 3e–f) were reported. The corresponding descriptions have also been added as follows.

Lines 85-88: *"Moreover, increasing trends in RHW frequency, duration and intensity were detected in 65.3%, 92.7% and 93.0% of basins, respectively, all higher than the corresponding proportions for AHWs (48.7%, 72.4% and 76.4%) (Extended Data Fig. 3e–f)."*

Second, the current feature importance analysis is based on XGBoost's built-in gain metric, which measures the average improvement in prediction accuracy contributed by each feature. While this approach is widely used and provides useful insights, it has a few known limitations. Given the growing adoption of SHAP (SHapley Additive exPlanations) in hydrological and climate impact studies, I would be curious to see whether the identified influential drivers remain roughly consistent under a SHAP-based framework. To ensure the robustness of your physical interpretations, please consider adding a supplementary figure with SHAP-based feature importance. This would strengthen the credibility of the driver attribution and make the interpretation more transparent.

Response: Thank you for the comment. As suggested, we have added a supplementary SHAP-based figure for comparison. The SHAP-based results are highly consistent with the original gain-based results in terms of both the dominant drivers and the category-level contributions (Extended Data Fig. 7), further supporting the robustness of our physical interpretations.

Specifically: **(1) the main influential drivers remain broadly consistent.** Under the SHAP framework, the primary predictors—including minimum air temperature, runoff, basin area, mean WT, and elevation-related variables—remain dominant, despite minor shifts in the ranking of secondary predictors. (2) **category-level contributions are highly aligned.** SHAP-based analysis attributes 59.6%, 20.8%, and 19.6% of total importance to climatic, topographic, and hydrologic variables, respectively. These values closely mirror the original gain-based results (i.e., 59.2%, 22.4%, and 18.3%), further consolidating our findings.

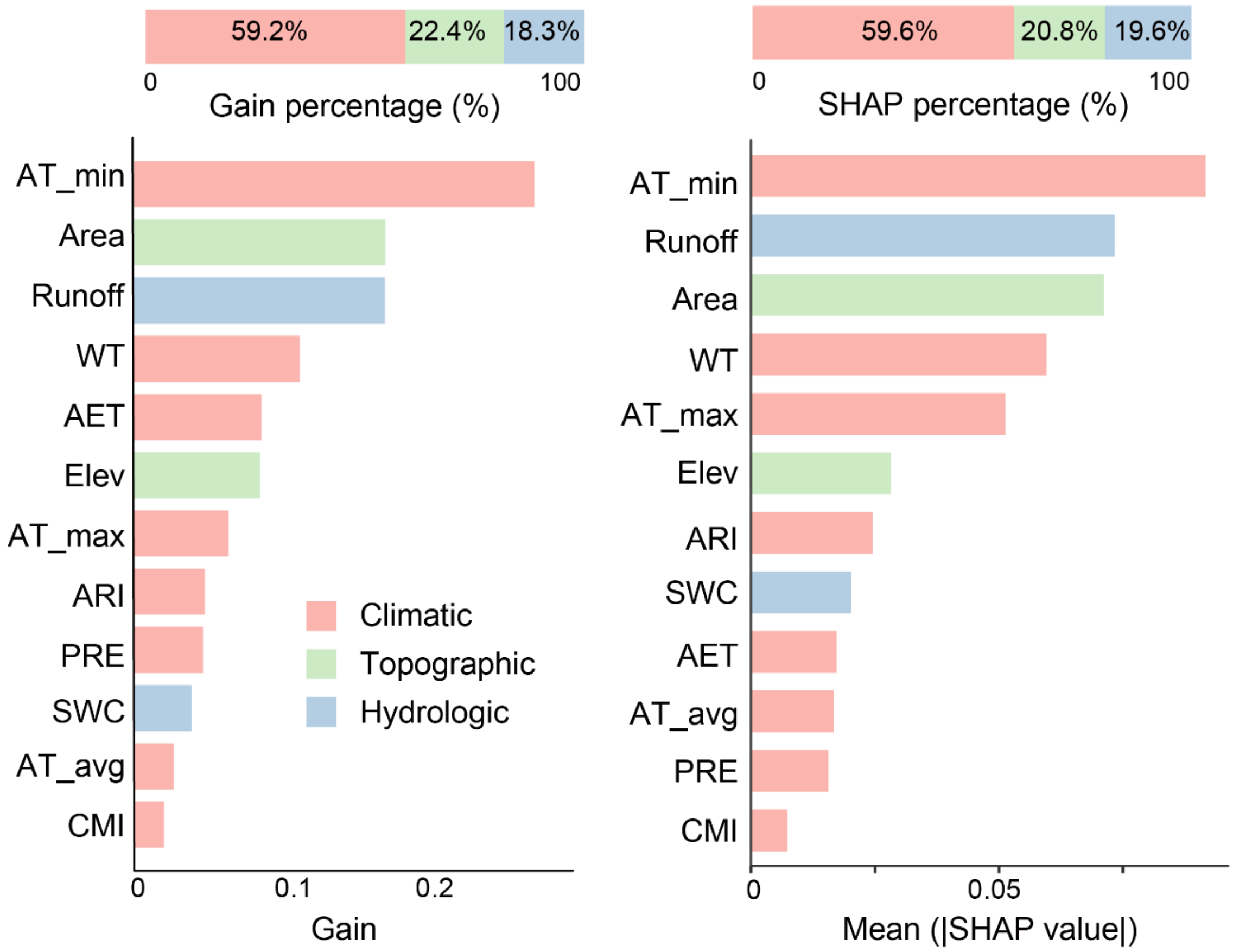

Fig. 3 (a) vs Extended Data Fig. 7. *Comparison of XGBoost gain importance and SHAP importance for the 12 selected predictors. Left, gain-based feature importance from Fig. 3 (a). Right, mean absolute SHAP importance from Extended Data Fig. 7. The stacked bars summarize the category-level contributions of climatic, geographic and hydrologic predictors.*

Given the above results, we have added the following to the revised text.

Lines 145–147: *"A supplementary SHAP analysis yielded broadly consistent patterns in both the dominant predictors and the category-level contributions, supporting the robustness of these attribution results (Extended Data Fig. 7)."*

The results suggest an elevation-dependent pattern in WT and DO changes. While the analytical framework is conceptually sound, the current presentation remains largely qualitative (e.g., phrases such as "substantial WT increases" and "smaller DO declines") and therefore lacks quantitative rigor. To strengthen this section, please add a more in-depth statistical analysis that explicitly quantifies the elevation dependence. In particular, I would recommend the authors to provide targeted quantification for the U.S. Rocky Mountains and the European Alpine regions—already highlighted in Figure 2—so readers can assess the magnitude and robustness of changes in these key

high-elevation hotspots. If necessary, consider using supplementary figures to visualize the elevation-dependent relationships more clearly.

Response: Thank you for the comment. As suggested, we have added supplementary figures (Extended Data Fig. 8g–j) to illustrate the elevation dependence of WT and DO changes, and further provided quantitative analyses for the U.S. Rocky Mountains and the Alpine region (Lines 206-207).

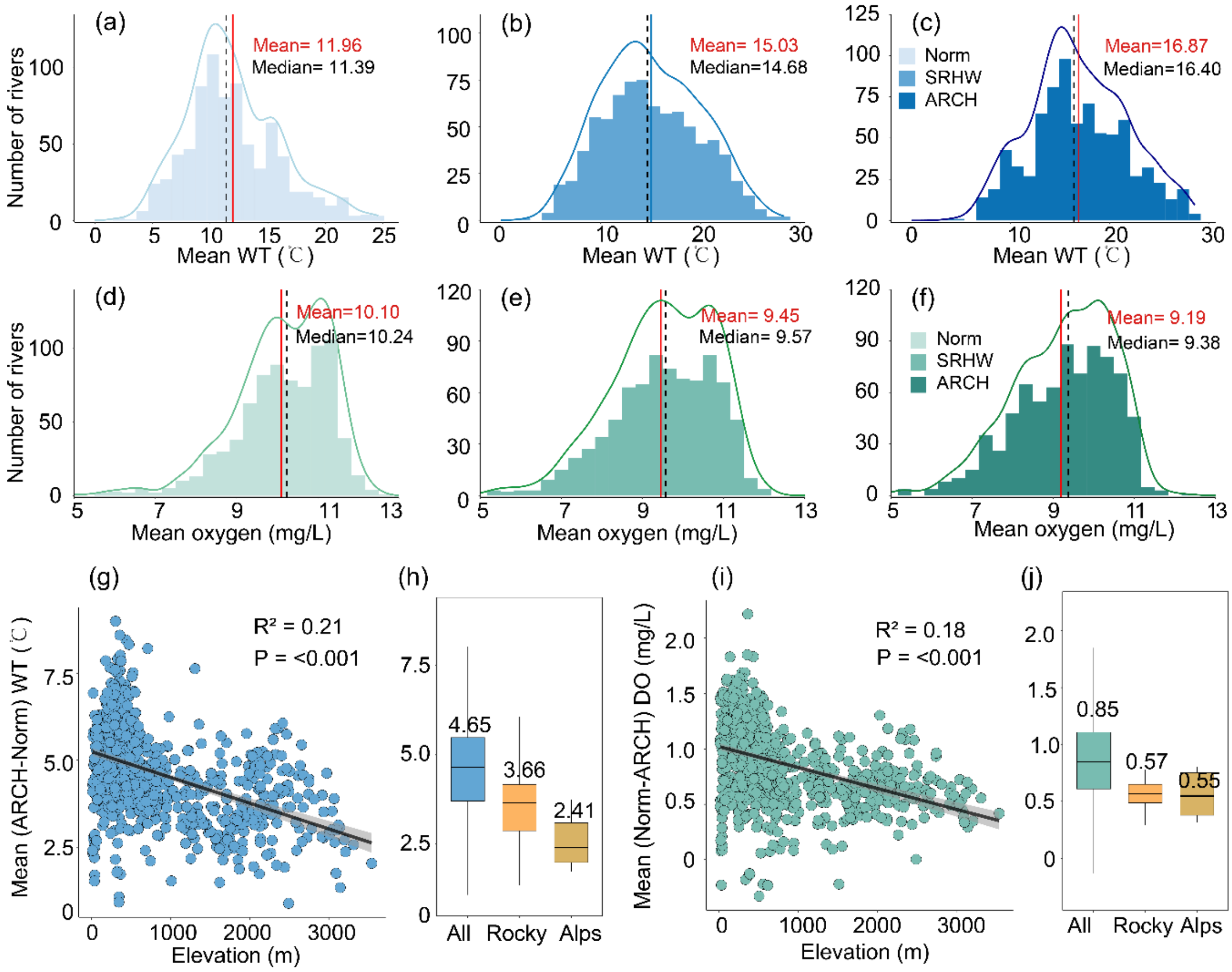

Extended Data Fig. 8 | WT and DO under heatwave conditions and elevation-dependent ARCH responses. *(a–c) Basin-level distributions of mean WT under Norm, SRHW and ARCH conditions. (d–f) Corresponding distributions of mean DO. Solid and dashed vertical lines indicate means and medians, respectively. (g, h) Elevation dependence and regional comparison of ARCH-induced WT changes. (i, j) Elevation dependence and regional comparison of ARCH-induced DO changes. Fitted lines show linear regressions with 95% confidence intervals; boxplots compare all basins, the Rocky Mountains and the Alpine region.*

We have added the following revisions:

Lines 203–209: *"Both ARCH-induced WT increases and DO declines exhibited modest but significant negative relationships with elevation ($p < 0.001$; Extended Data Fig. 8g, i), indicating stronger compound stress in lowland rivers. Specifically, ARCH-*

induced changes were smaller in the Rocky Mountains (+3.66 °C; -0.57 mg/L) and the Alps (+2.41 °C; -0.55 mg/L) compared to the average (WT: +4.65 °C; DO: -0.85 mg/L) (Extended Data Fig. 8h, j). However, the rapid increase of ARCH frequency suggests that mountain rivers may face growing risks under continued elevation-dependent warming."

The manuscript identifies a potentially important "tipping-point" transition (Figure 5 and S6): a notable regime shift where projected riverine heatwaves become less frequent but longer-lasting. This pattern may indicate that warming shifts river systems from episodic extremes toward chronic thermal stress, where individual events merge into longer episodes. However, the current manuscript does not sufficiently develop or contextualize this important finding. Please consider adding a dedicated discussion paragraph that 1) frame and discuss the tipping-point phenomenon within the broader context of climate and hydrology science; and 2) places the riverine results alongside the marine and lake heatwave literature. For example, whether similar projections have reported intensifying and lengthening heatwaves, potentially trending toward near-permanent conditions in other systems.

Response: We appreciate this constructive comment. As suggested, we have added a discussion paragraph to place this tipping-point behavior in a wider context.

Lines 289-300*: "Tipping points in river thermal dynamics. In Earth system, tipping points refer to critical thresholds where global warming triggers catastrophic and often irreversible changes to climate and hydrological systems[48]. Scientists warn that several tipping points may already be underway as global temperatures approach 1.5°C above pre-industrial levels, including melting ice sheets, coral reef collapse, and Amazon rainforest dieback[49,50]. Our findings suggest that riverine thermal systems may be approaching a similar transition under continued warming. The projected frequencies of AHWs, RHWs, and their compound ARCH events show clear tipping-point behavior (Extended Data Fig. 9) under the SSP5-8.5 scenario, with heatwaves becoming less frequent but increasingly prolonged and persistent. Similar patterns have also been reported in marine and lake systems[16,17]. Together, these findings suggest that continued warming may drive aquatic systems toward a regime shift, from episodic extremes to chronic thermal stress."*

Specific comments

1.There are several grammar errors. For example: Line 43, "Global high temperature records continues …" should be "continue"; Line 64, "RHWs is increasing faster".

Response: Thank you for bringing these to our attention. We have corrected the grammatical errors as suggested.

Line 39: *"Global temperature records **continue** to be broken, …"*

Lines 55-57: *"Recently, emerging evidence suggests that RHWs* ***are*** *increasing rapidly and may outpace atmospheric heatwaves in some regions[20–22]."*

2. In Line 57, there is an extra space before the citations "21, disrupt ecosystem metabolism 22,23, ".

Response: We have removed the extra space. Thanks.

3. Line 91, Figures 1 and S1 look clear and well presented. In the caption, please specify the "full study period". Also, I could not find the label "p < 0.001 (***)" in the figures.

Response: Thanks for the comment. We have clarified the "full study period" as follows.

Lines 101-103: *"Inset maps show the spatial distributions of mean annual RHW frequency, duration and intensity over the full study period, 1981–2019."*

We sincerely apologize that the labels were not clearly visible. We have marked them with blue "***" labels in Fig. 1.

4. Figure 2, it may be helpful to add the mean elevations of the Rocky Mountains and the Alps (e.g., in the figure panel or caption) to provide context and help readers interpret the patterns more easily.

Response: We appreciate this comment. Based on DEM data, we have added the mean and max elevations in the figure captions.

Lines 132-134: *"Dashed boxes mark the Rocky Mountains (mean and max elevations: 2037 and 4123 m) and the Alpine region (mean and max elevations: 2484 and 4193 m)."*

5. High-elevation mountains exhibit a clearly lower occurrence yet a higher growth trend of ARCH events (Figure 2). What about RHWs? Could this be related to elevation-dependent warming?

Response: This is a good question. RHWs in the high-elevation mountains (e.g., Rocky Mountains and the Alps) also show the same pattern (Fig. 1a–b). We have added the elevation-dependent discussion in the revised text.

Lines 125-129: *"A similar pattern is observed in RHWs within the Rocky Mountains and the Alps (Fig. 1a–b). Together, these consistent patterns suggest that mountainous rivers are emerging as fast-warming hotspots of compound heatwave risk, likely driven by elevation-dependent warming that causes high-altitude systems*

to warm faster than those at lower elevations[27,28].”

6. Line 248, this paragraph currently lacks a clear rationale for analyzing dissolved oxygen. Consider adding a brief sentence to justify DO as a key indicator of water quality.

Response: We appreciate this comment and have added a brief sentence as suggested:

Lines 173-174: “*Dissolved oxygen (DO) is a key indicator of water quality and aquatic ecosystem condition[29].”*

7. The manuscript occasionally uses inconsistent terminology for event frequency, such as “counts per year” and “events per year”. Check for consistency.

Response: We appreciate this comment. We have standardized the terminology as “events per year” throughout the manuscript.

Lines 109-110: *“ARCH events were widespread across the 796 river basins in the US and CE (Fig. 2a), occurring on average 1.77* ***events*** *per year.”*

Lines 123-125: “… *high-elevation regions (3000+ m) showed greater increases in both absolute (0.37* ***events****/decade) and percentage (128%/decade) ARCH frequency compared to low-elevation regions (<1000 m).”*

8. Line 346, Figure S6 shows an intriguing tipping point, where future riverine heatwaves tend to become less frequent but more persistent. Have similar phenomena been observed in other water systems?

Response: We thank the reviewer for this insightful comment. Yes, similar tipping-point phenomena have been reported in lakes and oceans. Accordingly, we have added a brief discussion to the revised manuscript, as follows.

Lines 297-300: *“Similar patterns have also been reported in marine and lake systems[16,17]. Together, these findings suggest that continued warming may drive aquatic systems toward a regime shift, from episodic extremes to chronic thermal stress.”*

9. Line 377, where is the supporting evidence for “~20% of RHWs occur in the absence of concurrent atmospheric extremes”?

Response: Thanks for the comment. We have included the supporting evidence (Extended Data Fig. 5) in the text.

Lines 254–255: “*Conversely, we observe that ~20% of RHWs occur in the*

absence of concurrent atmospheric extremes (Extended Data Fig. 5)."

10. Line 397, correct the typo "Although this responses is"

Response: We have corrected the typo. Thanks.

Lines 271: *"This **response** is…"*

11. Line 408, please clarify what "an additional 16%" is relative to.

Response: We appreciate this comment. We have clarified it as follows.

Lines 180–182: *"Relative to Norm conditions, WT increased by approximately 41% during ARCH and 25% during SRHW, corresponding to an additional 16% increase under compound events."*

12. The section of High prevalence of ARCH is interesting and insightful, especially in comparison with estuaries and marine systems. Have lake studies reported comparable ratios for atmospheric-lake compound heatwaves?

Response: We appreciate this insightful comment. We have performed an additional literature survey but found no relevant studies that report comparable compound ratios for atmospheric–lake heatwaves.

13. Before conclusion, it is necessary to add a few sentences outlining the key limitations of the data and the modeling approach.

Response: We agree and have added a few sentences as suggested.

Lines 286-288: *"Although long-term data availability and model capacity currently limit our assessment to the US and Central Europe, the continued expansion of global monitoring networks could refine ARCH risk assessments in future studies."*

14. Line 486, check format "25th, 50th, and 75th" with superscripts.

Response: Thanks for the catch. We have revised the format as suggested.

Line 449: *"…with the 25th, 50th, and 75th percentiles of testing NSE…"*

15. Line 508, the threshold definition for daily maximum air temperature is unclear.

Response: We appreciate this comment. The 90th percentile threshold refers to "the local, seasonal climatology". We have rephrased the sentence to clarify the threshold.

*Lines 469-471: "Consistent with previous studies, RHWs and AHWs were identified as periods of at least five and three consecutive days, respectively, during which daily river WT and daily maximum AT exceeded the 90th percentile of **the local seasonal climatology**[22,59]."*

16. Line 511, clarify the rationale for using the 11-day window, and provide supporting references (if available)?

Response: We appreciate this comment. As suggested, we have added the rationale and reference for using the 11-day window.

Lines 475-477: "The 11-day window is widely adopted in heatwave studies[17] to ensure robust sample size for percentile estimation while preserving the seasonal evolution of the climatology."

17. Line 580: It is not clear how the "197 regional attribute variables" were obtained and processed. Please add a brief description.

Response: We appreciate this comment. We have added further details as requested.

Lines 535-537: *"In this study, we compiled 197 regional attribute variables for 796 river basins from the HydroATLAS[64] database, including variables related to basin topography, climate, land cover, soil, geology, and anthropogenic influence."*

18. Line 581-582, clarify what kind of monthly data were removed?

Response: Thank you for this comment. We have clarified the data with examples.

Lines 537-538: *"After manually removing variables representing monthly data (e.g., tmp_dc_s07, aet_mm_s04),"*

19. For the XGBoost model, consider adding a supplementary table summarizing the tuned hyperparameters.

Response: Thanks for the comment. We have incorporated the optimized hyperparameters into the Methods section.

Lines 542-544: *"Grid search was subsequently used to optimize the XGBoost model with final hyperparameters as max_depth = 4, eta = 0.2, subsample = 0.7, colsample_bytree = 0.8, and nrounds = 68."*

Reviewer #2 (Remarks to the Author):

This manuscript investigates atmospheric–riverine compound heatwaves (ARCH) across 796 river basins in the US and Central Europe. The reported trends, especially the rapid growth of ARCH in high-elevation areas and their disproportionate impact on water quality (warming and deoxygenation), represent a potentially valuable contribution to the field. The topic is timely, relevant to the scope of the journal, and likely to be of interest to a broad readership. The manuscript is generally well organized and technically solid, and I would recommend a major revision to improve the rigor, clarity, and overall presentation of the manuscript.

Response: We sincerely thank the reviewer for encouraging assessment of our manuscript. All comments have been carefully addressed in the revised version. Please see below for our detailed point-by-point responses.

In Fig. 2c, the authors use a density plot to show the distribution of ARCH frequency across different elevation gradients. The overall pattern is generally clear, and it is possible to see that high-elevation mountain rivers above 3000 m tend to have lower ARCH frequency overall. However, I am concerned that the similar color palettes and overlapping curves may reduce the visual clarity of this result. This may also make it more challenging for readers to directly compare differences among elevation groups. The authors may wish to consider replacing this panel with a boxplot and adding significance testing among elevation groups, so that the group-level differences can be presented more clearly.

Response: Thank you for the constructive comment. We have replaced the density plot with a boxplot and added significance testing as suggested (Fig. 2c). It makes comparisons among elevation groups clearer.

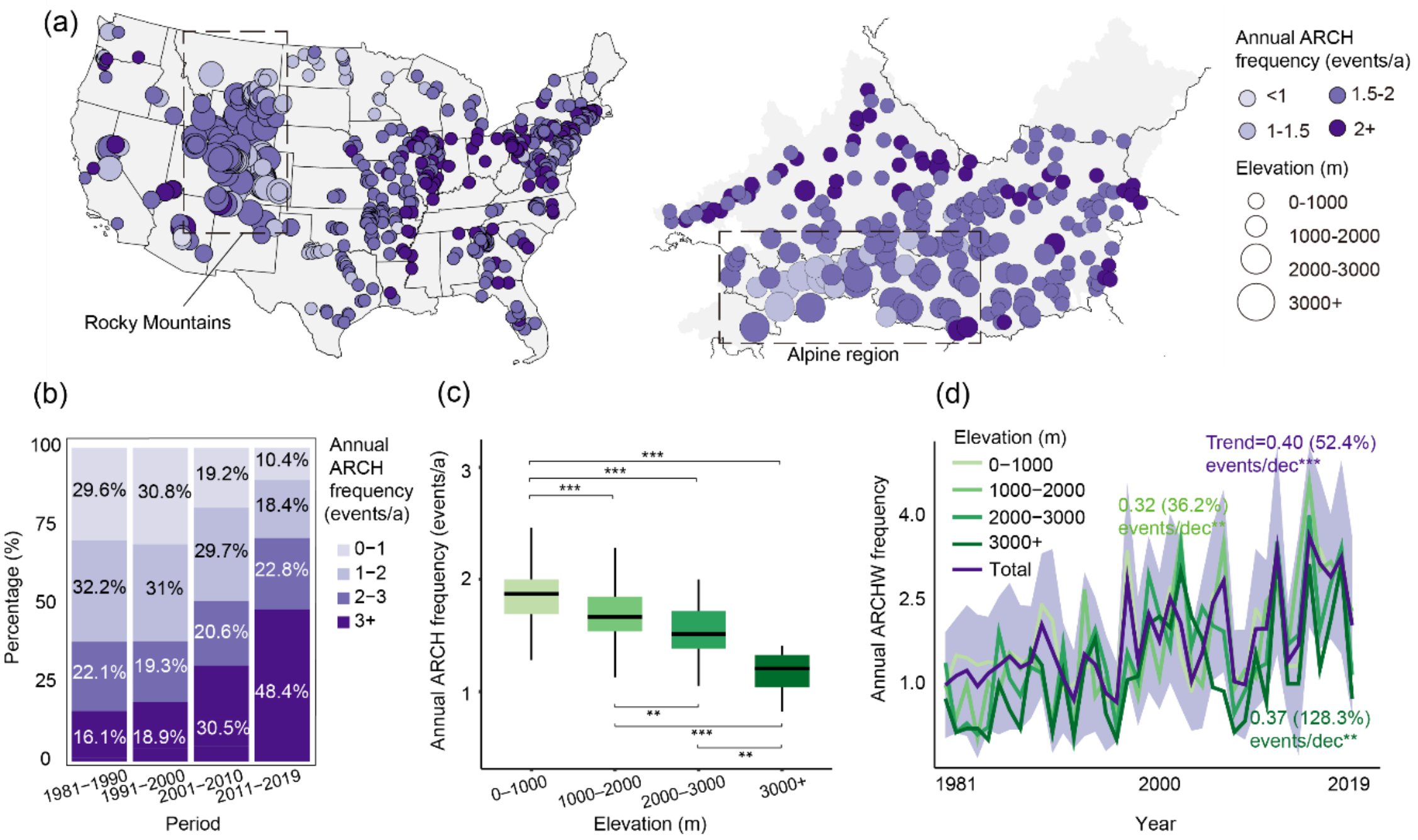

Fig. 2 | Spatiotemporal distribution of ARCH frequency. *(**a**) Mean annual ARCH frequency, with colour indicating frequency and symbol size indicating elevation. Dashed boxes mark the Rocky Mountains (mean and max elevations: 2037 and 4123 m) and the Alpine region (mean and max elevations: 2484 and 4193 m). (**b**) Percentage of rivers in four ARCH frequency classes across four periods. (**c**) Boxplots of basin-level mean annual ARCH frequency across elevation classes, asterisks indicate significant differences. (**d**) Annual trends in ARCH frequency from 1981 to 2019 for all basins and across elevation classes.*

2. The manuscript reports that, relative to normal conditions, compound heatwaves are associated with an approximately 41.0% increase in WT and a 9.8% decline in DO. While these numbers and figures effectively illustrate the impact of compound events, the pronounced disparity in the magnitude of these responses deserves further elaboration. I recommend that the authors draw more on the literature concerning riverine dissolved oxygen dynamics and their controls, and expand the analysis from the perspective of multiple interacting processes, in order to better explain this asymmetry. Such discussion would strengthen the mechanistic interpretation of the results.

Response: We appreciate this insightful suggestion. We have expanded the discussion to explain the asymmetric response in WT and DO.

Lines 186-188: *"The larger relative increase in WT (41%) indicates a tight coupling between atmospheric and water temperature, while the smaller decline in DO (9.8%) suggests additional controls beyond temperature forcing[30], such as light, flow, and anthropogenic influences."*

3. The authors use SSP2-4.5 and SSP5-8.5 to project future heatwave changes, which is an appropriate selection of scenarios. However, the manuscript currently provides only limited context regarding the socioeconomic narratives and climate forcing levels represented by these pathways. I therefore recommend that the authors include a concise briefing at the beginning of the relevant section to define these scenarios. Specifically, clarifying that SSP2-4.5 represents a "Middle of the Road" pathway with intermediate forcing, while SSP5-8.5 denotes a high-emission, fossil-fuel-intensive development scenario, would be beneficial.

Response: Thanks. We agree and have added a brief description as suggested. Given the word limit and the broad use of these scenarios, we kept this definition concise.

Lines 210-211: *"Future thermal risks are assessed under both the intermediate (SSP2-4.5) and high (SSP5-8.5) emission scenarios."*

4. The discussion notes that approximately 20% of RHWs occur independently of AHWs. This is an intriguing result and may suggest a certain degree of decoupling between water and air temperature. The authors currently attribute this pattern primarily to anthropogenic regulation, which is a plausible explanation. However, natural controls should also not be overlooked. Beyond human intervention, the intrinsic thermal buffering capacity of river systems, driven by processes such as groundwater-surface water interactions (hyporheic exchange), riparian shading, and the thermal inertia of large water bodies, may play a critical role in modulating this decoupling. I therefore encourage the authors to expand this section by incorporating relevant literature on these natural factors, so as to provide a more balanced discussion.

Response: We appreciate this constructive comment. As suggested, we have expanded the discussion to include natural factors.

Lines 255-258: "*This decoupling likely reflects the combined influence of natural and anthropogenic controls. Natural processes, including groundwater–surface water interactions[37], riparian shading[38], water-column thermal inertia[39] can modulate the coupling between AT and WT.*"

5. Other minor comments regarding language, formatting, and notation are as follows:

(1) Line 43: "Global high temperature records continues to be broken" contains a subject–verb agreement error.

Response: We have corrected the error.

*Line 39: "Global temperature records **continue** to be broken, …"*

(2) Line 53: This sentence appears to contain a grammatical error.

Response: We appreciate this comment. We have corrected the error.

Lines 47-48: "As climate warming continues, AHWs are projected to further escalate[8,9], making heatwaves a heightened feature of future climate risk."

(3) Line 395: "underling" appears to be a typo and should likely be "underlying".

Response: Thanks for the catch. We have removed the word "underlying"

(4) The expression used for ARCH frequency is not fully consistent, with "frequency", "counts", and "occurrences" used interchangeably.

Response: We appreciate this comment. The terminology has been standardized throughout the manuscript, and "frequency" is now used consistently to describe

ARCH frequency.

*Lines 113-114: "A temporal trend shows that high-**frequency** ARCH events expanded substantially over time,"*

*Line 262-263: "Our analysis attributes ~60% of variation in ARCH **frequency** to climate change, with AT_min emerging as the dominant driver (Fig. 3)."*

(5) The caption of Fig. 2 contains interpretive statements that may be more appropriate for the main text rather than the figure legend.

Response: We appreciate this comment. We have moved the interpretive statements to the main text.

Lines 123-125: *"… high-elevation regions (3000+ m) showed greater increases in both absolute (0.37 events/decade) and percentage (128%/decade) ARCH frequency compared to low-elevation regions (<1000 m)."*

(6) The use of "MWT" and "WT" is not fully consistent; the main text mostly uses WT, whereas Fig. 3 uses MWT.

Response: Thanks for the catch. For consistency, we have changed "MWT" to "WT" in Fig. 3.

(7) The legend in Fig. 2b lacks units.

Response: We appreciate this comment. We have added the missing units (i.e., events/a) to Fig. 2b.

(8) The definition of the shaded areas in Fig. 5 is not consistent across the text and caption. In the main text, the shaded area is described as "se" (Line 323), whereas in the figure it is labeled as "std". This should be clarified.

Response: Thanks for the comment. We have standardized the notation to "std" across the text and figure captions.

*Lines 223-224: "Annual maximum AT under historical and future scenarios, with shaded bands showing model **std**."*

*Lines 226-227: "Annual maximum WT under historical and future scenarios, with shaded bands showing model **std**."*

(9) The formatting of date ranges is inconsistent, with some instances using an en

dash and others a hyphen.

Response: We have checked the manuscript thoroughly and standardized the formatting of all date ranges throughout the revised manuscript. Thanks.

*Lines 67-69: ", reconstructing continuous daily records (**1981–2019**) across 796 rivers in the US and Central Europe (CE) (Extended Data Fig. 1, 2)."*

*Line 79: "…between **1981–1990** and **2010–2019** were larger than…"*

(10) The notation for elevation thresholds is inconsistent, including ">3000 m", "3000 m+", and "3000+ m". A standardized format should be used throughout.

Response: We appreciate this comment. We have standardized all text and figure labels to "3000+ m" throughout the manuscript.

*Lines 121-122: "It is noteworthy that although high-elevation river basins (**3000+ m**) have a lower average annual ARCH frequency,"*

*Lines 123: "high-elevation regions (**3000+ m**) showed greater increases in …"*

(11) Line 191, the expression "improved model performance by 59.2%" may not be fully accurate. The authors may wish to revise this to "contributed to model performance" or similar wording.

Response: We agree and have revised this expression accordingly. The revised text is as follows:

Lines 142-143*: "Among these, climatic factors (primarily AT, WT, evapotranspiration, and precipitation) contributed 59.2% to model performance."*

(12) Line 397: "this responses is modulated" contains a grammatical error. Please correct it.

Response: We appreciate this comment. We have corrected this error.

Line 271: *"This **response** is…"*

(13) Spacing and notation for numbers, units, and percentages should be checked throughout the manuscript. For example, in Line 1004, "25th, 50th" should be formatted as superscripts.

Response: Thanks. As suggested, we have checked and revised the spacing and notation for numbers, units, and percentages throughout the manuscript.

Line 449: *"25th" and "50th"*

(14) Reference 43 appears incomplete and lacks full bibliographic information. References 3 and 116 refer to the same paper.

Response: We appreciate this comment. We have substantially reduced and revised the reference list, including the removal of the previously incomplete Reference 43, and removed duplicated references, including the former Reference 116. We have also carefully checked all references to avoid incomplete entries and duplicate citations.

(15) Supplementary Figure S8, it is not clear whether the reported NSE values refer to the training or testing period. This should be clarified.

Response: We appreciate this comment. The reported NSE values refer to the testing period. We have clarified it in the revised text and figure caption.

Lines 447-448: *"Across all river basins, the LSTM model achieved a median **testing NSE** of 0.72 for DO, …*

*Lines 449-450: ", with the 25th, 50th, and 75th percentiles of **testing NSE** being 0.90, 0.95, and 0.97, respectively."*

*Lines 451-452: "we selected four representative basins with **testing NSE** values corresponding to the 25th, 50th, 75th, and 100th percentiles."*

*Lines 636: "Extended Data Fig. 1 | **Testing** performance …"*

Reviewer #2 (Remarks on code availability):

The authors have provided a well-documented code repository that includes a comprehensive README file. The instructions for environment setup and execution are clear, and the code is runnable and able to reproduce the main figures presented in the manuscript.

More broadly, I believe the code will be a valuable resource for the community, particularly for researchers working on riverine heatwaves and related hydroclimatic extremes.

Response: We thank Reviewer #2 for the positive comments on the code availability and reproducibility. We are glad that the repository is clear and may serve as a useful resource.

*******************END*******************

Response to Review Comments

Referee comments:

Reviewer #1 (Remarks to the Author):

The revised manuscript, "Threefold increase in atmospheric–riverine compound heatwaves under climate change," addresses an important and timely knowledge gap by investigating the occurrence, trends, drivers, and water-quality consequences of compound atmospheric–riverine heat extremes. The revision responds well to the editor's key concerns by providing stronger evidence for recent changes and underlying mechanisms, and by better articulating how these compound extremes may push river systems toward critical thresholds under future climate warming. These additions further strengthen the manuscript's novelty, mechanistic depth and broader relevance to climate-impact science.

Overall, I have carefully reviewed both the revised manuscript and the detailed response to comments. The authors have done a thorough and meticulous job in addressing the comments raised by the editor and the two reviewers, including those related to the figures, methodology, and discussion. The revised manuscript has therefore been substantially improved, and I am very supportive of its publication in its current form.

Response: We very much appreciate the reviewer's constructive, insightful, and thorough assessment of our revised manuscript. These valuable comments have substantially improved the manuscript.

Reviewer #2 (Remarks to the Author):

The authors have addressed my comments well, I have no other comments

Reviewer #2 (Remarks on code availability):

The code is great.

Response: We sincerely thank the reviewer for the constructive assessment and positive evaluation of our revised manuscript and code. The reviewer's helpful comments have greatly improved the quality and clarity of our work.
